# A predictive modeling approach for cell line-specific long-range regulatory interactions

**DOI:** 10.1093/nar/gkv865

**Published:** 2015-10-10

**Authors:** Sushmita Roy, Alireza Fotuhi Siahpirani, Deborah Chasman, Sara Knaack, Ferhat Ay, Ron Stewart, Michael Wilson, Rupa Sridharan

**Affiliations:** 1 Department of Biostatistics and Medical Informatics, University of Wisconsin-Madison, Madison, WI, USA; 2 Wisconsin Institute for Discovery, 330 N. Orchard Street, Madison, WI, USA; 3 Department of Computer Sciences, University of Wisconsin-Madison, Madison, WI, USA; 4 Department of Genome Sciences, University of Washington, Seattle, WA, USA; 5 Morgridge Institute for Research, Madison, WI 53715, USA; 6 Genetics & Genome Biology Program, Hospital for Sick Children (SickKids) and Department of Molecular Genetics, University of Toronto,Toronto, ON, Canada; 7 Department of Molecular Genetics, University of Toronto, ON, Canada; 8 Department of Cell and Regenerative biology, University of Wisconsin, Madison, WI 53715, USA

## Abstract

Long range regulatory interactions among distal enhancers and target genes are important for tissue-specific gene expression. Genome-scale identification of these interactions in a cell line-specific manner, especially using the fewest possible datasets, is a significant challenge. We develop a novel computational approach, Regulatory Interaction Prediction for Promoters and Long-range Enhancers (RIPPLE), that integrates published Chromosome Conformation Capture (3C) data sets with a minimal set of regulatory genomic data sets to predict enhancer-promoter interactions in a cell line-specific manner. Our results suggest that CTCF, RAD21, a general transcription factor (TBP) and activating chromatin marks are important determinants of enhancer-promoter interactions. To predict interactions in a new cell line and to generate genome-wide interaction maps, we develop an ensemble version of RIPPLE and apply it to generate interactions in five human cell lines. Computational validation of these predictions using existing ChIA-PET and Hi-C data sets showed that RIPPLE accurately predicts interactions among enhancers and promoters. Enhancer-promoter interactions tend to be organized into subnetworks representing coordinately regulated sets of genes that are enriched for specific biological processes and *cis*-regulatory elements. Overall, our work provides a systematic approach to predict and interpret enhancer-promoter interactions in a genome-wide cell-type specific manner using a few experimentally tractable measurements.

## INTRODUCTION

The human genome encodes thousands of regulatory DNA sequence elements that control spatial and temporal patterns of gene expression (1). A major mechanism by which regulatory elements such as enhancers act on a target gene is through chromosomal looping (2–6) where a distal enhancer is brought close to a target gene in three-dimensional space. Such long-range regulatory interactions are emerging as important determinants of tissue-specific expression (4,7,8) and for interpretation of regulatory variation (9,10). Experimental techniques for detecting these interactions such as Chromosome Conformation Capture (3C) (5) and its variants (4C, 5C (11), Hi-C (12–14), Capture-Hi-C (15), DNase-Hi-C (16)) and ChIA-PET (17,18) are quickly maturing and differ in the resolution and the genomic coverage of regions interrogated. However, multiple components of the transcription machinery facilitate these interactions, including histone modifications for activating and poised transcription (19,20), transcription factors (21) and components of the cohesin complex (22). As such, the principles by which such elements act upon their target genes to drive tissue-specific expression and the interplay of long-range gene regulation with other one-dimensional regulatory signals such as transcription factor occupancies or chromatin modifications are not well understood. Hence, there is a need for integrative approaches that can leverage multiple types of regulatory genomic data sets to provide a genome-wide characterization of enhancer-promoter interactions across multiple cell lines.

Approaches that integrate diverse regulatory genomic data sets have served as useful complements to experimental approaches in gene regulation studies, e.g. for identifying enhancers (23–25) and for reconstructing transcriptional regulatory networks (26–28). Recently, computational approaches for predicting interactions among enhancers and promoters have also been developed. One strategy has been to use correlation of DNase I footprints or chromatin marks across multiple cell lines for each potential enhancer and promoter pair (24,25,29). More recently, approaches that combined H3K4me1, CTCF and mRNA levels (9) and supervised learning approaches that used ChIA-PET data sets to train a classifier (30), improved on correlation-based approaches. However, there are several issues that need to be addressed for predicting enhancer-promoter interactions in a cell line-specific manner.

The first issue is to determine the most informative measurements to predict such interactions in a new cell type or time point, where such interactions might be difficult to measure experimentally. Recent comparison of experimentally detected enhancer-promoter interactions across multiple cell lines has shown that the interactions tend to be cell line-specific (11,18,31). One idea is to apply a classifier trained on one cell line to predict interactions in the new cell line. However, it is not clear which cell line's classifier or what data sets need to be measured in the new cell line to predict such interactions. A related issue is to determine whether additional regulatory genomic data sets other than those that have been commonly used for enhancer-promoter interactions (e.g. CTCF (9), DNase I (29), H3K4me1 (9,30), RNA-seq (9,30), H3K4me3, H3K27ac (30)) have additional value. A third issue is that current approaches to predict enhancer-promoter interactions have built one predictor for all cell lines examined (9,29,30). Building a classifier for each cell line is likely to be more sensitive to cell line-specific interactions and can discriminate between different types of cell line-specific interactions. For example, interactions could be obliterated in one cell line either because the enhancer (or promoter) is no longer active in the cell line, or the enhancer and promoter remain active but do not interact (e.g. because of chromosomal domains (4)).

In this paper, we make two contributions. First, we present a new computational method RIPPLE (Regulatory Interaction Prediction for Promoters and Long-range Enhancers) to predict interactions between distal enhancer elements and target genes in multiple cell lines. RIPPLE is based on a supervised machine learning framework that uses interactions detected from 5C experiments in a particular cell line as a training set and a small number of genomic data sets selected from the ENCODE collection of regulatory genomic data sets (32). To select a minimal set of data sets for RIPPLE while ensuring good predictive power across multiple cell lines, we developed a hybrid feature selection strategy that uses both Random Forests feature importance measure (33) and multi-task learning (34) with Group Lasso (35). Classifiers trained on these selected features have high predictive performance on both 5C data and Hi-C data. Second, we develop an ensemble approach to predict interactions between new genomic regions and in new cell lines with no available 5C data sets. Our ensemble approach can robustly identify interactions for new cell lines, especially when the best training cell line classifier is not known upfront.

Using our ensemble approach we generated genome-wide enhancer-promoter interactions for four cell lines with 5C data and a new cell line (HepG2) for which we did not have 5C data. Examination of our predictions from multiple cell lines shows that cell line-specific interactions are between enhancers and promoters that are active in two cell lines, but they interact in only one of the cell lines. Our genome-wide interactions are significantly enriched for experimentally measured interactions (e.g. from ChIA-PET and high resolution Hi-C) providing external validation of our predictions. In addition to being associated with known regulatory proteins (e.g. CTCF, RAD21) and activating chromatin marks (e.g. H3K4me3, H3K9ac), our predictions also implicate novel factors (CMYC, H3K36me3, H3K79me2) that have not been studied extensively for establishing long-range interactions. Overall our work provides a systematic way to predict cell line-specific enhancer-promoter interactions using a minimal set of data sets in multiple cell lines and offers important insights into the interplay between architectural proteins, general transcription factors and histone modifications for establishing these interactions.

## MATERIALS AND METHODS

There are three important components to RIPPLE: (i) Learning a single cell line-specific classifier, (ii) Determining the minimal number of data sets for predicting interactions in multiple cell lines, (iii) Ensemble learning to predict interactions in new cell lines.

### Learning a single cell line-specific classifier for predicting enhancer-promoter interactions

**Figure 1.**
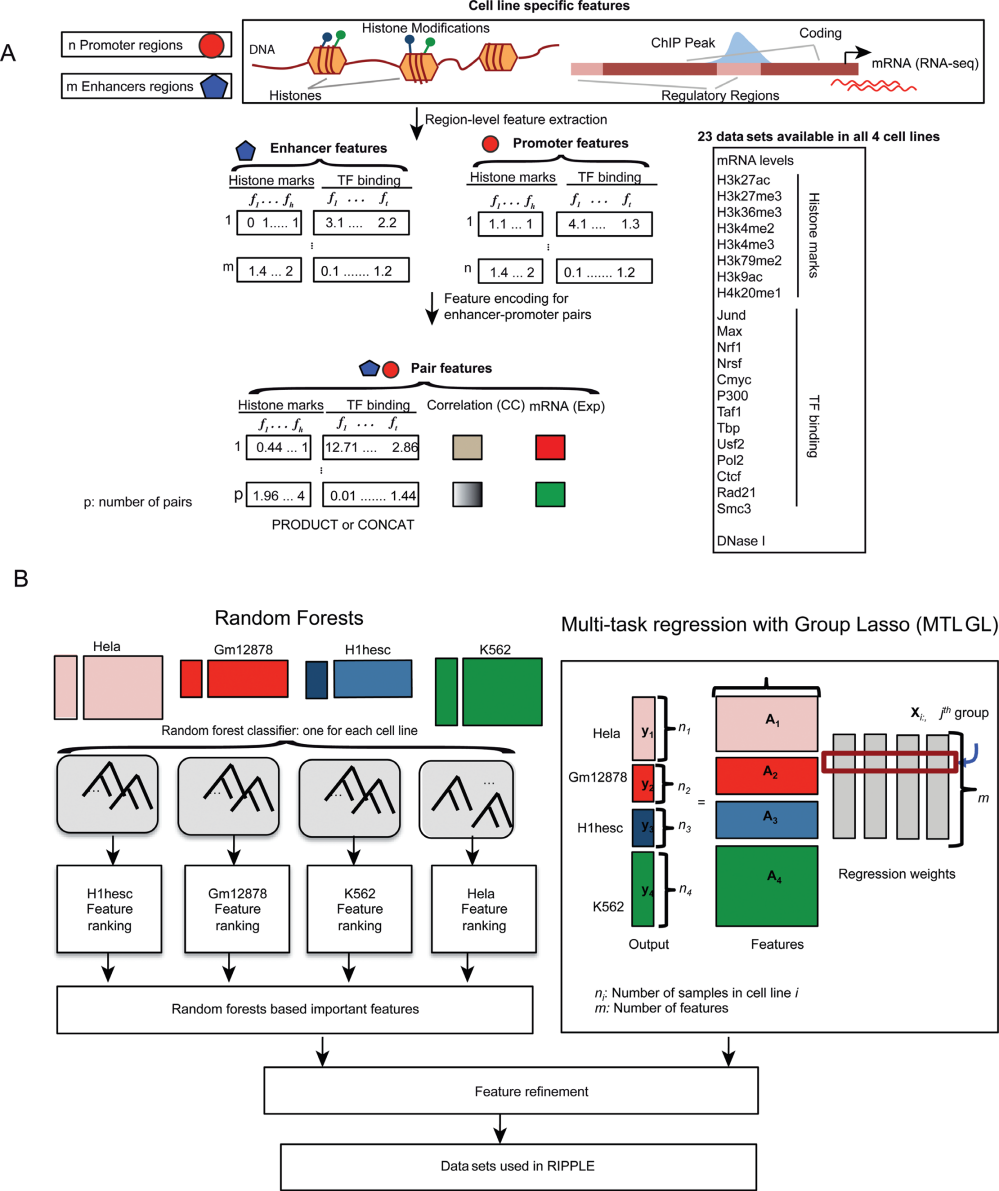
RIPPLE classification framework for predicting cell-line specific enhancer-promoter interactions. The two main stages of building RIPPLE's cell line-specific classifier: identification of appropriate feature encoding and selection of minimal data sets. (**A**) Encoding an enhancer-promoter pair for a classifier. ChIP-seq (for general transcription factors and histone modifications), DNase I and RNA-seq data sets measured in a given cell line provide feature values for an enhancer or a promoter genomic region. The feature values can be continuous (negative log of *P*-value of signal enrichment, gene expression levels) or binary (presence absence of a particular peak). To represent an enhancer and promoter pair to a classifier, we use two strategies: CONCAT and PRODUCT. In CONCAT, we concatenate the feature vector associated with an enhancer with the feature vector associated with a promoter. In PRODUCT, we use the product of the feature value on the enhancer and the promoter to specify the feature value of the pair. We also use the correlation of the signal values on the enhancer and promoter as an additional feature. (**B**) Our hybrid approach to identifying the minimal feature set for building cell line-specific classifier. We train cell line-specific Random Forests on labeled 5C data and use standard feature importance measures (out-of-bag error) in Random Forests to rank the features. In parallel, we use a multi-task learning with Group Lasso to perform joint feature selection across all four cell lines. The intersection of both approaches is used as input for the feature refinement step, where we remove and add individual, pairs or triplets of features guided by the correlation of the features. The output of this step gives us a minimal set of data sets for RIPPLE.

RIPPLE is based on a predictive learning framework where the goal is to train a binary classifier on examples of interactions and non-interactions using features derived from various regulatory genomic data sets (e.g. ChIP-seq data sets for histone modifications, transcription factor occupancies, RNA-seq, Figure 1). To build a predictive model, we need to decide on a feature representation of an enhancer-promoter pair, generate positive and negative sets, and determine the best learning algorithm for our problem.

#### Feature representation

To extract features for RIPPLE's classification algorithm, we used data sets from the ENCODE project for four cell lines: K562, Gm12878, Hela S3 (Hela) and H1 Embryonic Stem cell (H1hesc), learning a separate classifier for each cell line. Because our end goal was to build a classifier that works across multiple cell lines, we focused on 23 data sets that were measured in all four cell lines (Figure 1). These 23 data sets included 8 histone marks, 15 transcription factors, DNase I and RNA-seq. ChIP-seq data sets are represented as a collection of peak calls that have two genomic coordinates of chromosome start and stop. We used the peak files generated by the ENCODE consortium (36,37) (http://ftp.ebi.ac.uk/pub/databases/ensembl/encode/integration_data_jan2011/byDataType/peaks/jan2011/histone_macs/optimal/ for histone marks and http://ftp.ebi.ac.uk/pub/databases/ensembl/encode/integration_data_jan2011/byDataType/peaks/jan2011/spp/optimal for transcription factors). SMC3 ChIP-seq data for the H1hesc cell line was obtained from the Gene expression omnibus (GSM897119) and the MOSAiCS peak calling algorithm was applied to call peaks (38).

We represented an enhancer or promoter region as a real or binary vector, each dimension corresponding to one of the 23 genome-wide data sets (Figure 1). A promoter had an additional feature representing the mRNA level of the gene associated with the promoter. For the binary case, a ChIP-seq or DNase I data set was represented as 1 or 0 depending upon whether a particular peak had ≥1bp overlap with the regulatory region. In the real vector case, we used the −log(*P* value) of the signal enrichment. If multiple peaks with different values overlapped a region we took the larger value. Expression levels were always represented as continuous features.

To generate a feature vector for a pair of enhancer promoter regions we used two strategies: CONCAT and PRODUCT (Figure 1A, Supplementary Figure S1). In the CONCAT case we concatenated the feature vectors of the *n*-dimensional feature vectors of the enhancer and promoter regions to obtain a feature vector of size 2*n*, where *n* includes everything other than the RNA-seq data set. In the PRODUCT case, each enhancer-promoter pair was represented using an *n*-dimensional vector, each dimension representing the product of the corresponding values in the enhancer or the promoter feature vector. For the case of real feature values, we added a small correction of 0.01 to each dimension to avoid setting a feature value of the pair to zero, if one of the regions did not have a feature value. Finally, for each pair, we included two additional features, the Pearson's correlation of the *n* signals (same for binary or real) associated with an enhancer to signals associated with the promoter of a pair; and the RPKM expression level of the gene associated with the promoter. To assess the performance of a specific feature encoding we used the Area Under the Precision-Recall curve (AUPR), which measures the tradeoff in the precision and recall of predictions as function of classification threshold, estimated with 10-fold cross validation (Supplementary Figure S1). AUPR was computed using AUCCalculator (39). We trained and tested a Random Forests classifier for all four cell lines using the different feature encodings. We find that the best AUPRs were given by the CONCAT feature compared to the different versions of the PRODUCT features. We also evaluated the utility of correlation and expression by combining the CONCAT or PRODUCT features with expression only (CONCAT+E), correlation only (CONCAT+C) and correlation and expression (CONCAT+C+E). The CONCAT feature with expression and correlation (CONCAT+C+E) was the overall best performing feature representation. Because the difference between continuous and binary features was not significant, we used the binary features because it makes cross-cell line comparisons less sensitive to the tree rules learned by a Random Forest in a training cell line. Based on these results, we represented an enhancer promoter pair using the CONCAT+C+E feature set.

#### Positive and negative set generation

RIPPLE uses Carbon Copy Chromosome Capture Conformation (5C) derived interactions as a positive data set from Sanyal *et al*. (11). The 5C data set statistics are in Table 1. Sanyal *et al*. used 5C to detect interactions among pairs of targeted genomic loci in manually selected and random ENCODE regions representing 1% of the human genome. They designed a collection of forward and reverse primer pairs for these genomic regions. Reverse primers were designed for regions overlapping a transcription start site (TSS), while forward primers were for non-TSS regions. In our approach, we defined promoters as a genomic region that had a TSS (Gencode v10) or a TSS was <2500 bp away. We defined distal elements as those that are at least 2500 bp away from a TSS, thus being necessarily distal from the target promoter. In addition we filtered the enhancer promoter regions such that they had at least one non-zero feature values. To generate the negative set, we sampled from the non-interacting primer pairs while controlling for the distance between interacting pairs. This was important because the tendency for an enhancer to interact with a promoter depends upon the distance between them. To ensure that the positive and negative examples exhibited the same distance distribution, we binned all measured primer pairs into 10 Kbp bins. For any pair of interactions that is in the positive set that is assigned to a bin, *b_i_* , we sample uniformly at random from the set of non-interacting pairs from the same bin *b_i_*. In both the positive and negative set we considered pairs of enhancers and promoters that were in <1 MB apart and that had features associated with them. We trained classifiers on a balanced data set but evaluated performance on the same sized negative set (1 times) as well as on a larger negative sets that was 10 times the size of the positive set (10 times, Supplementary Figure S2A). In all cases the classifiers are significantly better than random (Supplementary Figure S2A), however for the 10 times negative set the AUPR was lower. Here a random classification prediction was obtained by shuffling the probabilities of interactions among the pairs. Many of the ‘false positives’ that are not detected in the 5C data set can be true interactions. To provide additional support to these predictions we compared the difference in contact counts in the positive and negative sets in the 5C data, and compared this to predicted positives and negatives in the 1 times and 10 times negative sets and found that at all classification probability thresholds of >0.5 the contact counts of our predicted positives are significantly higher than negatives (Supplementary Figure S2B, 2C).

**Table 1.** Number of enhancers, promoters and enhancer-promoter interactions in 5C training data sets for RIPPLE

Cell line	Enhancers	Promoters	Interactions
K562	327	859	877
Hela	301	757	765
Gm12878	471	227	476
H1hesc	196	334	337

#### Learning algorithms for predicting enhancer-promoter interactions

We considered two types of approaches as the main predictive learning algorithm for RIPPLE: Random Forests approach and regularized regression approaches. Random Forests are powerful ensemble learning approaches that have been shown to have very good generalization performance (33). We trained Random Forests (RF) on different feature encodings using 500 trees. Random Forests were trained and tested with 10-fold cross-validation where we trained the classifier on training data from 9 out of 10 folds and tested on the left out data. The performance of the RF classifier was examined using AUPR. The AUPR was calculated using the prediction probability of an enhancer-promoter pair when it was part of the test set.

In addition to Random Forests we also applied two regularized regression based classification approaches: (i) L1-penalized linear regression (LASSO), and (ii) L1-penalized logistic regression (GLMLASSO). These L1-regularized regression approaches are powerful predictive models while at the same time perform feature selection to learn a ‘sparse’ model (40) where the regression coefficients of many features are set to 0 thus performing model selection. L1-regularized regression approaches require the selection of the regularization parameter, λ, which is typically selected based on cross-validation. We used the MATLAB statistics package implementation of L1-regularized linear regression (lasso) and regularized logistic regression (glmlasso). Prior to using the linear regression approach we converted the 0-1 labels to −1 and +1, although the linear regression classifier on the 0-1 labels had similar AUPRs. As with the Random Forests, we performed 10-fold cross-validation to assess the performance of each classifier. In each fold of cross validation (CV) we performed another round of 5-fold cross validation to select λ that minimized prediction error in the test set of that CV fold. Once the best λ is selected for a fold, we re-learned the regression weights using this λ.

### Building a minimal classifier by combining Random Forests and Group Lasso-based Multi-task learning

**Figure 2.**
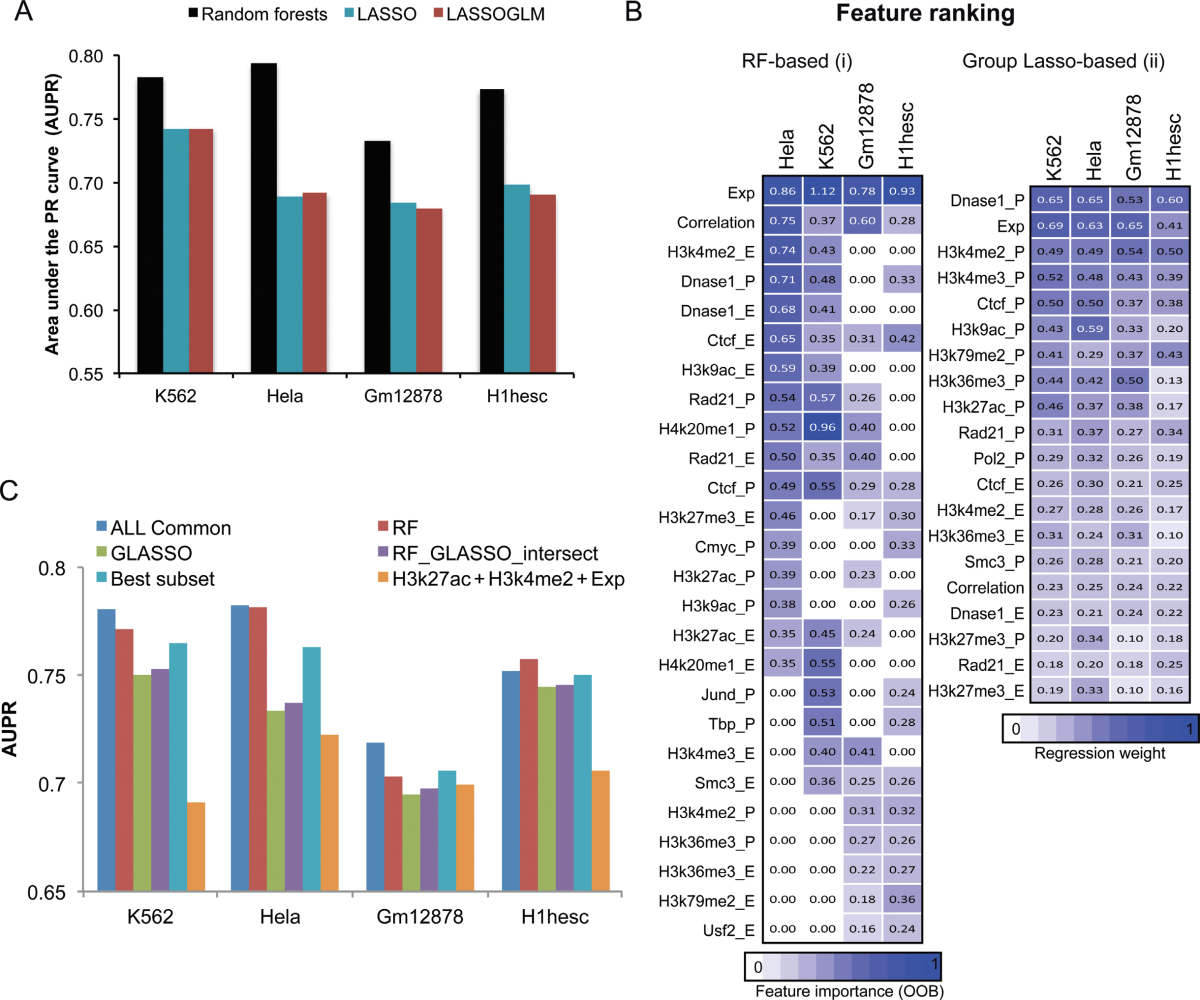
Evaluation of different feature encodings and classification algorithms for enhancer-promoter interaction prediction. (**A**) Area Under the Precision-Recall curve (AUPR) values for all four cell lines and the three classification approaches tested. These approaches include the Random Forests classifier, a regularized linear regression approach (LASSO) and a regularized logistic regression approach (LASSOGLM). The higher the bar the better the particular classification approach. (**B**) Top selected features using Random Forests and Group Lasso. For Random forests the feature importance is the out of bag error when the feature is included in the top 20, and 0 otherwise, and for Group Lasso the feature importance is the absolute value of the regression coefficient. (**C**) AUPRs on different combinations of data sets: ALL Common: all 23 data sets, GLASSO: 13 data sets selected by Group Lasso, RF: 17 data sets selected by Random Forests feature ranking, RF_GLASSO_intersect: 12 data sets in the intersection of data sets selected by Group Lasso and Random Forests, H3k27ac+H3k4me2+Exp: 3 data sets including H3K27ac, H3K4me2 and RNA-seq based gene expression levels.

To determine the most important features and that have good predictive power for all cell lines, we combined Random Forests feature selection with features selected using a structured sparsity approach based on Multi-task learning and Group Lasso (described below). Our motivation for using this strategy is as follows: Random Forests are powerful classifiers for the task at hand, however, they do not offer model selection. That is, given *n* features to a RF classifier, it will learn a predictive model that uses all *n* features. On the other hand, sparse learning approaches such as those based on Lasso can do model selection by setting some coefficients of features to 0. However, such a model does not perform as well as a Random Forests approach (Figure 2A). Furthermore, independently training a classifier on each cell line would not necessarily identify the same set of features across cell lines, making it difficult to assess how well a classifier would generalize to new cell lines. We therefore used a hybrid approach for determining the most important data sets that is informed both by the sparsity-imposing regularized regression framework as well as by RF feature importance and performance measures across all cell lines studied. First, using a regularized multi-task learning framework, we identified features that were important for all four cell lines. Second, using the RF-based feature importance ranking, we found important features that were in the top 20 in at least two of the four cell lines. We then used the intersection of the features deemed as important by our multi-task learning framework and Random Forests feature ranking as the initial set of features. We then refined this feature set while considering features that were ranked as important by Random Forests but not by our sparse learning method.

We used a multi-task learning framework because we had four classification problems, one for each cell line, and we needed a feature to be selected based on its utility across all four classification problems. Multi-task learning is a popular machine learning approach that aims to simultaneously solve multiple learning problems to share information between the learning problems (34). In our problem setting the different learning problems are the classification tasks, one for each cell line. Each classification task is defined as $Y_c=\beta _c\mathbf {X}_c$, where *c* ranges from 1 to 4 for our four cell lines and our multi-task learning approach aims to select features that are useful for all cell lines. $Y_c$ is the vector of $m_c$class labels, $\mathbf {X}_c$ is a matrix of feature vectors, and β_*c*_ represents the regression weights for the *c*^th^ cell line. In order to select the features important for all cell lines, we use the Group Lasso framework (35). Group Lasso is a particular type of structured sparsity approach that enables one to exploit group structure among the predictive co-variates in the model while imposing sparsity constraints. In our problem the group structure is imposed by the requirement of selecting features that are important in not one but all four cell lines. Specifically, let $\mathbf {B}$ denote a *N_common_* × *K* matrix, where *N_common_* is the number of features common to all cell lines. Let β_*c*_ denote the *c*^th^ column of $\mathbf {B}$. The ‘group’ is defined by each row of the $\mathbf {B}$ matrix and corresponds to the set of regression coefficients {β_1_(*j*), ⋅⋅⋅, β_*K*_(*j*)} for the *j*^th^ feature. In Group Lasso, the regularization takes the form of an L1/L2 norm, and the objective function is defined as

\begin{equation*} \widehat{\mathbf {B}}=\sum _{c=1}^{K}||Y_c-\beta _c\mathbf {X}_{c}||_{2}^{2} + \lambda ||\mathbf {B}||_{1/2} \end{equation*}

The L1/L2 norm for $\mathbf{B}$ is defined as $\sum _{j=1}^{N_{common}}||\mathbf {B}_{j}||_2^{2}$, where $\mathbf {B}_j$ is the *j*^th^ row of $\mathbf{B}$. The effect of this norm is it imposes sparsity at the level of entire rows, setting them to 0.

To perform multi-task Group Lasso for our problem we used the Sparse Learning with Efficient Projections (SLEP) package (http://www.yelab.net/software/SLEP/). Specifically we used the mtLogisticR function with 10-fold cross-validation. In each fold we applied the mtLogisticR function of the multi-task classification problem. Final features were selected such that they were in at least 8 of the 10 folds of cross-validation. Such features were ranked based on the sum of the absolute value of the regression weights across all cell lines followed by selecting the top 20 features.

Feature importance in a Random Forests classifier was computed using the ‘Out of bag’ error. This computes feature importance as the decrease in classification accuracy for out of bag examples when the feature is permuted in the training set. The feature was selected if it was among the top 20 features in two of the four cell lines. Furthermore, a data set (e.g. CTCF) would be included if it was important either at the enhancer or at the promoter region.

After obtaining important features from both the Random Forests classifier and multi-task Group LASSO, we performed an additional step of feature refinement starting with the intersection set of both Group Lasso and Random Forests based approach. Our feature refinement procedure was guided by the extent to which the features were correlated (Supplementary Methods, Supplementary Figure S3). We considered subsets of data set combinations based on their correlation structure to first remove additional features that would not significantly reduce performance, followed by another iteration of adding data sets (Supplementary Figure S4). For example, CTCF, SMC3 and RAD21 were highly correlated in all four cell lines, and we evaluated the performance when removing any of these individually or in combination (CTCF-SMC3, CTCF-RAD21, SMC3-RAD21). The feature refinement step resulted in a minimal set of data sets that were used to train an RF classifier that served as the main predictive model of RIPPLE.

### Evaluation of RIPPLE on Hi-C data

In addition to training classifiers on 5C data, we assessed the performance of RIPPLE on chromatin interactions identified by the Hi-C experiments of Rao, Huntley *et al*. (13) (GEO Accession GSE63525) for the Gm12878 and K562 cell lines. For the Gm12878 cell line, we used the ‘Gm12878_combine’ data set, which was a combination of two separate replicates of the Gm12878 experiment.

Using their Hi-CCUPS method, Rao, Huntley *et al*. identified significant intrachromosomal interactions between regions of uniform size. They did so separately for three different resolutions of regions: 5, 10 and 25 Kbp. To create a data set for RIPPLE, we divided the genome into 5 Kbp regions. As in the 5C case, we defined promoters as regions that were within 2500 bp of a TSS (Gencode v10). We defined ‘distal’ (putative enhancer) regions as those that were further than 2500 bp from a TSS. We then removed any regions that had no feature data (using the same feature data sources as for 5C). Next, we used the significant interactions from the original publication to create a set of true positive interactions. Specifically, each true interaction consisted of a promoter region and a distal region that appeared on opposite sides of a significant interaction at any of the three available resolutions.

To define a balanced set of negative pairs (each consisting of one 5 Kbp promoter region and one distal region), we employed a similar procedure as for 5C data (described above). In addition to controlling for distance between the regions, we also controlled for expression of the promoter in the cell line. This step was important as the space of possible negative pairs was dominated by non-expressed promoters, while most promoters in positive pairs had non-zero expression. Specifically, a negative drawn to match a positive for which the promoter region had non-zero expression was also required to have non-zero expression. This procedure identified a total of 2761 positive promoter-distal pairs for Gm12878 and 6649 for K562.

We assessed the performance of RIPPLE based on AUPR on Hi-C data in three experiments. First, we performed ten-fold cross-validation within each cell line (Gm12878 and K562). For each fold, we trained a Random Forests classifier of 500 trees on training data from 9 out of 10 folds and tested on the hold out fold. Second, we assessed the performance of RIPPLE to predict Hi-C interactions between cell lines by training on examples of one cell line and testing on examples of the other cell line. Third, we measured the ability of RIPPLE to generalize across the Hi-C and 5C platform on the same cell line. For each of K562 and Gm12878 cell lines, we trained a Random Forests classifier on all the data from one platform and tested on data from the other platform.

### Ensemble approach to predict interactions in new cell lines

To generate predictions in a new cell line, we employed an ensemble approach to combine predictions from multiple cell lines. We considered two approaches that used predictions from cell line-specific classifiers, Percentile and Spectral Meta Learner, and a third baseline approach that pools the training examples from all training cell lines to predict interactions in a test cell line. The percentile approach is described as follows: suppose we have *K* different classifiers, *K* = 4 in our setup. For the *i*^th^ test pair of interest, we obtain classification probabilities from each classifier, *p_ij_*, 1 ≤ *j* ≤ *K*. We convert each *p_ij_*, into percentile ranks and take an average of these ranks. The Spectral Meta Learner approach uses the correlation between predictions of different classifiers (41). This approach automatically weights the different predictions based on the absolute values of the leading eigen vector and was shown to outperform a majority voting based ensemble (41). Specifically, let Σ be a *K* × *K* covariance matrix of the prediction probabilities and let $\mathbf {e}$ be the eigen vector of Σ corresponding to the largest eigen value. The prediction of the ensemble is then the dot product, $\mathbf {p}_i*\mathbf {e}$, where $\mathbf {p}_i=\lbrace p_{i1},\cdots , p_{iK}\rbrace$. These values were then used to rank each prediction. Our third baseline approach simply combined the data from different cell lines. Specifically, to predict interactions for cell line *l*, we merged the examples from all cell lines *k* ≠ *l*, trained a classifier on this larger set of examples, and then predicted interactions for pairs in cell line *l*.

### Generating genome-wide predictions

Our genome-wide predictions were applied to a set of ‘universal’ enhancer and promoter regions so that the same input set of genomic regions were considered in each cell line. To create these enhancer regions we took cell line-specific enhancer predictions from ENCODE (42) for the Gm12878, H1hesc, K562, Hela S3 and HepG2 cell lines. These predictions were generated by reconciling the genome segmentations from ChromHMM and Segway (42). For each cell line, we obtained regulatory regions predicted as enhancers by using one of two tags ‘E’ for predicted enhancer and ‘WE’ for additional weak enhancers at http://ftp.ebi.ac.uk/pub/databases/ensembl/encode/integration_data_jan2011/byDataType/segmentations/jan2011/hub/ genomeSegmentation.html. We obtained the enhancers from each of the four cell lines and generated a universal set of enhancers by combining all the enhancers. If two enhancers were adjacent to each other or overlapped, we merged them into a single region. We excluded enhancers that were <200 bp in length. To define promoters we considered ±2500 of TSS annotations from Gencode v10. In total, we had 52 065 regions that we called promoters and 216 042 regions called enhancers. The number of regions reduced in a cell-line specific manner after overlapping them with the data sets in our minimal classifier: K562: 117 707, Hela: 110 585, Gm12878: 98 064, H1hesc: 106 007, HepG2: 115 834 enhancers, and K562: 30 173, Hela S3: 28 304, Gm12878: 24 721, H1hesc: 27 359, HepG2: 27 136 promoters. We considered all pairs of enhancers and promoters that were <1 MB apart and that had features associated with them.

### Computational validation of genome-wide predictions from RIPPLE

We used two measures to validate the genome-wide predictions from RIPPLE. The first was based on the difference in Hi-C contact of predicted interactions in the 90% compared to interactions predicted to be below the 10% percentile. We obtained Hi-C data for H1hesc cell line from Dixon *et al*. (43). This data was normalized using the Iterative Correction and Eigen value decomposition method (ICE) (44). We binned the normalized data into 10 Kbp resolutions. Next for each predicted interaction, we found a corresponding bin pair where one region of the interaction overlapped with one of the bins of the pair, and the other end of the interaction overlapped with the other bin pair and used the contact count for this bin pair for our predicted interaction. We did this analysis for the predicted interactions from RIPPLE and the predicted interactions from the PRESTIGE (9) and IM-PET (30) methods for the H1hesc cell line.

We also evaluated the genome-wide predictions based on their ability to recapitulate interactions measured using experimental approaches that are complementary to 5C. Specifically, we obtained ChIA-PET data sets (17,18), and a high-resolution Hi-C data set from IMR90 (14). We mapped these interactions onto the genome-wide regions by requiring one member of an interaction to overlap with one of the enhancers in the genome-wide data set and the other region to map to a promoter in our genome-wide data set. We defined fold enrichment as the ratio of observed overlap fraction of interactions, $n_1/n_2$, to expected overlap fraction of interactions, $m_1/m_2$ and is computed as $\frac{n_1/n_2}{m_1/m_2}$. Here $n_1$is the number of interactions in the predicted set of interactions that overlap with an interaction in an experimental data set. $n_2$ is the total number of interactions in the predicted data. $m_1$is the total number of interactions in the experimental data set. $m_2$ is the total number of interactions possible.

### Clustering and subnetwork analysis

We applied K-means clustering with 10 multiple restarts to cluster the enhancers and promoters in the 90% percentile genome-wide network. We used the Euclidean distance between the feature vectors for an enhancer or promoter for clustering. We next assigned the pattern of chromatin modifications, architectural proteins, DNase I and DNA binding proteins observed in a cluster to a cluster ID from 1 to 5. We compared these patterns across cell lines manually to uniformly assign the same ID for a pattern across cell lines. Thus cluster *i* in one cell line would have as close as possible a pattern as cluster *i* in another cell line. We repeated the procedure for both the enhancer and promoter clusters.

To test whether enhancer cluster *i* (EC_*i*_) interacted significantly with a promoter cluster (PC_*j*_), we used a Hypergeometric test of enrichment and a fold-enrichment. Let the total number of enhancer-promoter interactions be the background $T$. We defined $n$ to be the total number of interactions associated with PC_*j*_, and $m$ to be the total number of interactions leaving from EC_*i*_ and $p$ to be the number interactions from EC_*i*_ to PC_*j*_. We ask using the Hypergeometric test the probability of observing $p$ or more interactions of $n$ interactions to come from EC_*i*_ given that there are $m$ interactions in all. Fold enrichment is calculated as $\frac{p}{n}/\frac{m}{T}$. We observed a fold enrichment that was slightly greater than 1 to be highly significant, and, therefore used fold enrichment as our measure of comparison.

To identify subnetworks we performed a connected components analysis on the network of enhancer-promoter interactions. To examine whether the promoter clusters and these subnetworks are associated with biological functions we tested them for enrichment based on the Hypergeometric test (Benjamini Hochberg corrected *P*-value <0.05). We tested each set of genes (either from a subnetwork or promoter cluster) for enrichment of curated gene sets from Gene Ontology, MSigDB curated pathways (45), which includes KEGG, REACTOME and BIOCARTA, and MSigDB motifs.

### Implementation and availability

Feature set generation was done using a custom C++ program. Regularized linear and logistic regression and Random Forests were applied using the MATLAB statistics toolbox. Multi-task Group Lasso was performed using the SLEP learning package (http://yelab.net/software/SLEP/). C++ program for feature set generation and MATLAB scripts used to train the Random forests on 5C and Hi-C and to apply RIPPLE on new enhancer promoter pairs are available at our supplemental website http://pages.discovery.wisc.edu/∼sroy/ripple/index.html. The code is also available at https://bitbucket.org/roygroup/ripple/downloads. RIPPLE classifier predicts the probability whether an enhancer interacts with a promoter. The higher the probability the more likely is an enhancer to interact with the promoter. Predicted genome-wide interactions with their probabilities for each of the five cell lines studied in this work are available online at our supplemental website http://pages.discovery.wisc.edu/∼sroy/ripple/index.html. These interactions can be downloaded as tab-delimited files as well as queried using a region or gene of interest.

## RESULTS

### Accurate prediction of enhancer-promoter interactions require a combination of histone marks, CTCF, cohesin and a general transcription factor

To predict new enhancer-promoter interactions for previously unstudied genomic loci and cell types and to systematically identify important determinants of long-range distal regulatory interactions, we developed RIPPLE. RIPPLE uses a Random Forests (RF) classification algorithm as its predictive model, which performs significantly better than linear or logistic regression-based algorithms (Figure 2A). RIPPLE is trained on features derived from 11 data sets that we identified by refining the features obtained from two complementary feature selection approaches, namely, Multi-task Group Lasso and RF feature importance (Materials and Methods). These data sets include architectural proteins (CTCF), cohesin (RAD21), activating marks of transcription (H3K4me2, H3K27ac, H3K9ac), marks associated with active gene bodies and elongation (H3K36me3, H4K20me1), repressive mark (H3K27me3), open chromatin (DNase I), a general transcription factor (TBP) and gene expression level. The Multi-task Group Lasso method identified 13 data sets as important for enhancer-promoter interaction prediction: CTCF, SMC3, RAD21, DNase I, expression, H3K27ac, H3K27me3, H3K36me3, H3K4me2, H3K4me3, H3K79me2, H3K9ac, RNA PolII and RAD21 (Figure 2B). The features selected by Multi-task Group Lasso were almost a complete subset of features selected by Random Forests, with the exception of RNA PolII (Figure 2B). Using Random Forest feature importance measure, we additionally found H4K20me1, and general transcription factors such as USF2, TBP, CMYC and JUND. An RF classifier trained on the 13 Group Lasso selected features (Figure 2C, GLASSO), gave a reduced performance compared to our full set of 23 data sets (Figure 2C, ALL Common, one-sided T-test <0.05), whereas, an RF classifier trained on the 17 data sets selected using the RF-feature selection had no significant difference (one-sided T-test <0.18) suggesting the six data sets that were not selected by RF as important are not essential for predicting enhancer-promoter interactions (Figure 2C). An RF classifier trained on the 11 data sets feature set has very comparable performance (Figure 2C, Best subset), as the classifier trained on the full set of 23 data sets (Figure 2C, ALL column), demonstrating that these data sets specify the minimal data sets needed for accurate enhancer-promoter interaction predictions.

A common way to link enhancers to promoters is to use canonical enhancer (e.g. H3K27ac, H3K4me1) and promoter marks (H3K4me3) together with mRNA levels of a candidate gene (30). We asked if the data sets selected by RIPPLE had greater accuracy in addition to what would be obtained using H3K27ac (our 23 input data sets did not include H3K4me1) at the enhancer and H3K4me3 and gene expression levels at the promoters. We found that these three data sets were very informative by themselves alone but they had much lower performance than the data sets used by RIPPLE (Figure 2C, orange bar). These results suggest that the additional data sets used in RIPPLE that include architectural proteins, chromatin marks and a general transcription factor (such as TBP) are important for accurately predicting enhancer-promoter interactions and can improve performance over known marks associated with enhancer-promoter interactions.

### RIPPLE identifies both shared and cell line-specific interactions and additionally discriminates between region and interaction specificity

**Figure 3.**
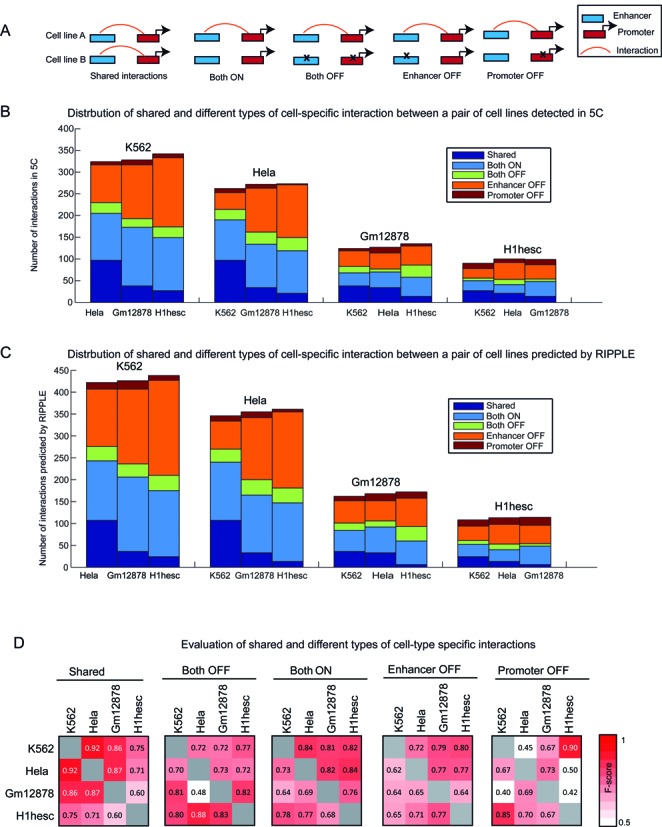
Characterization of different types of cell line-specific interactions. (**A**) Shown are the different statuses of an interaction comparing a pair of cell lines, (**A**) and (**B**). These statuses can be, ‘shared’, or cell-line specific. Cell-line specific interactions in turn can be grouped into ‘Both OFF’, ‘Enhancer OFF’, ‘Promoter ON’ and ‘Both ON’. (**B**) Shown are the relative proportions of the different interaction types in the 5C data when comparing each cell line to one of the other three cell lines. (**C**) Shown are the relative proportions of the different interaction types in the RIPPLE predicted interaction networks. The relative proportions of different types of interactions are similar between RIPPLE and 5C. (**D**) Shown is the agreement (as measured by F-score values) of interactions in different categories predicted based on RIPPLE and based on 5C. The higher the F-score the greater the agreement.

Having determined the key data sets needed for building RIPPLE, we next turned our attention to cell line-specific interactions. For two cell lines A and B, an interaction is said to be specific to cell line A if the interaction is detected only in cell line A, but not in B. There are four possible states for an interaction seen in cell line A and not in B (Figure 3A): (i) Both OFF: both enhancer and promoter are OFF/inactive in cell line B (we define ON and OFF below), (ii) enhancer-OFF promoter-ON: the enhancer is OFF in cell line B, but the promoter is ON, (iii) enhancer-ON promoter-OFF: the enhancer is ON, but the promoter is OFF in cell line B, (iv) BOTH ON: both enhancer and promoter are ON in cell line B, but the regions do not interact in cell line B. We defined an ON enhancer for a particular cell line to be that which had a peak for one or more of these signals in that cell line: DNase I, H3K27ac, H3K9ac. These three signals are commonly used to define enhancers or enhancer-like regulatory elements (29,46). We defined an ON promoter if the promoter had H3K4me3 (a promoter specific mark for active transcription, (46)) or DNase I, but not H3K27me3, a mark associated with repressive action on gene expression. We first obtained the distribution of different types of interactions for each pair of cell lines in the 5C data (Figure 3B). We found that the majority of cell line-specific interactions are enhancer-OFF promoter-ON, or of type BOTH ON, where both regions are ON but they do not interact. While the significant contribution of OFF enhancers to cell line-specific interactions is expected, the non-trivial fraction of cell-line specific interactions despite an enhancer being ON was surprising.

We next examined these configurations among the predicted interactions. We defined a predicted set of interactions for these 5C regions using a classification probability of interaction >0.5 (determined by a significant difference in contact counts between predicted interactions and non-interactions, Supplementary Figure S2B). We categorized predicted interactions from each pair of cell lines into the five categories in Figure 3A and found a similar distribution of shared and different types of cell line-specific interactions as in the 5C case with the Both-ON and Enhancer-OFF categories constituting the majority of the cell-line specific interactions (Figure 3C). Finally, we compared the categorizations of the RIPPLE predicted and true 5C interactions for each pair of cell lines using F-score, defined as the harmonic mean of precision and recall of the predicted and 5C-measured interactions in each category (Figure 3D). We found that RIPPLE is able to accurately predict interactions in shared and the two largest categories of cell line- specific interactions for all pairs of cell-lines compared, with an F-score of 0.6–0.9 for the SHARED interactions and an F-score of 0.69–0.72 for Enhancer OFF categories. RIPPLE was also able to correctly infer predictions for most pairs of cell lines in the Promoter OFF and Both OFF categories. This suggest that RIPPLE can accurately capture both shared as well as cell line-specific interactions.

### RIPPLE approach generalizes to both Hi-C and 5C technologies

To examine whether our enhancer-promoter interaction prediction strategy and the data sets selected using 5C data generalize to interactions detected using other 3C experimental techniques, we applied our RIPPLE approach to high-resolution Hi-C data recently made available for two cell lines Gm12878 and K562 (13). These Hi-C data were sequenced sufficiently deeply to be able to detect interactions at resolution comparable to the 5C platform.

First, we performed 10-fold cross-validation within each cell line (Gm12878 and K562). Based on AUPR from 10-fold cross-validation within the K562 (0.776) and Gm12878 (0.845) cell lines, the RIPPLE features perform well on the Hi-C data (PR curves shown in Supplementary Figure S5A). These values are comparable to what we obtained on the 5C data suggesting our prediction framework is applicable to Hi-C interactions as well. Next, we measured the ability of RIPPLE to generalize between the Hi-C and 5C platform on the same cell line. For each of K562 and Gm12878, we trained a Random Forests classifier on all data from one platform and tested on the other. The classifiers perform comparably in both directions on both cell lines (AUPRs: K562 5C to Hi-C 0.643, Hi-C to 5C 0.631; Gm12878 5C to Hi-C 0.687, Hi-C to 5C 0.614, Supplementary Figure S5B). This suggests that the features used in RIPPLE and our general predictive learning approach can generalize between different 3C platforms.

### Predicting enhancer-promoter interactions in new cell lines: Different classifiers were able to predict interactions in a new cell line to different extents

**Figure 4.**
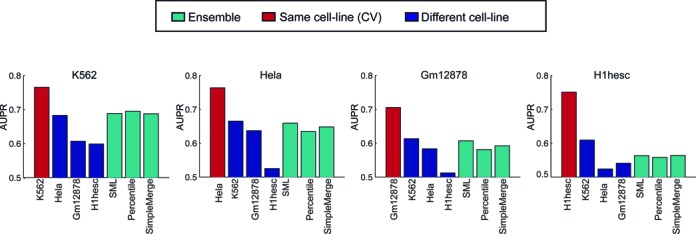
Predicting interactions in new cell lines. Ability of RIPPLE to recover interactions in a new cell line on which the classifier is not trained on. The red bar corresponds to the best case performance, i.e. when using cross-validation in the same cell line. The red bars correspond to the AUPRs when using a classifier from a different cell line. The cyan bars correspond to the ensemble based predictions: Percentile, SML (Spectral Meta Learner), SimpleMerge are the three types of ensemble approaches we used to pool information from different cell lines to predict interactions in a new cell line not used for training.

Our analysis so far demonstrates the feasibility of predicting new regulatory interactions among enhancers and promoters in cell lines with available 5C data. We next asked if we could extend this approach to predict interactions in new cell lines where such 5C data does not exist. This would require us to train a classifier on one cell line and predict interactions in a different cell line. To address this we applied the RIPPLE classifier trained on one cell line to enhancer-promoter pairs from a different test cell line. We evaluated the quality of the predictions in the test cell line by comparing to the AUPR obtained under cross-validation when trained on the test cell line (Figure 4, Same-cell line CV). We found that we were able to recover a significant fraction on the performance for some cross-cell line comparisons. For example, a classifier trained on the Hela cell line had an AUPR of 0.68 when applied to the K562 cell line (which was 89% of the AUPR obtained based on 10-fold cross-validation (CV) on the K562 cell line). Similarly, a classifier trained on the K562 cell line was able to accurately predict interactions from the Gm12878 cell line (87% of the AUPR obtained on Gm12878 CV classifier). However, there was considerable variation in the ability to predict interactions from a cell line (e.g. H1hesc interactions were hard to predict from either Hela and Gm12878). The cross-cell line performance behavior is recapitulated in the Hi-C data as well. In particular, we find that a classifier trained on Gm12878 can predict K562 interactions (AUPR 0.725), and a classifier trained on K562 can predict Gm12878 interactions (AUPR 0.816, Supplementary Figure S5C). Although in our panel of four cell lines, we found that K562 was the best predictor of different cell lines, this is likely because it has the largest training data set for both Hi-C and 5C. While one would expect a cell line to better predict interactions for a second cell line with similar transcriptional and epigenetic properties, the four cell lines used in this study are too few to determine this property reliably. Hence, determining the specific cell line that can be used to predict interactions in a new cell line is not straightforward.

One approach to addressing the issue of determining which classifier should be used for a new cell line is to combine predictions from all available cell lines using an ensemble approach. Ensembles are less prone to over fitting and are commonly used for different classification approaches. To this end we examined the quality of predictions for a new cell line if predictions from all other cell line classifiers would be used. We combined predictions from different cell lines using three approaches (Materials and Methods), (i) a commonly used average of percentile ranks of predictions (Figure 4, Percentile), (ii) a recent approach, Spectral Meta learner (SML) proposed by Parisi *et al*. (41) (Figure 4, SML), which uses the eigen vector of the covariance matrix of prediction probabilities to obtain a weighted sum of predictions from the individual classifier predictions, (iii) a generic classifier that combines examples from all other cell lines to predict interactions in a new cell line (Figure 4, SimpleMerge, e.g. for H1hesc, we used a classifier trained on data pooled from K562, Gm12878 and Hela). We found that all ensemble approaches performed comparably (Figure 4, cyan bars), but were at par with the best performing cross-cell line classifier and better than the worst performing cross-cell line classifier. This suggests that the ensemble represents a safe choice for a new cell line. We used the percentile-based approach to build our ensemble for our subsequent experiments as it is easy to extend to new cell lines and was better than the SML ensemble on genome-wide interactions (Supplementary Figure S6). In a genome-wide setting, an ensemble based classifier is better at recovering interactions experimentally measured by ChIA-PET and high resolution Hi-C compared to a CV classifier for that cell line as we describe next.

### RIPPLE inferred genome-wide maps are corroborated with experimental data sets

We applied RIPPLE trained on each of the four cell lines to generate genome-wide predictions of enhancers from ENCODE (42) and promoters (defined by ±2500bp of the TSS) for five cell lines. Our cell lines included the four cell lines with 5C data, as well as a new cell line, HepG2, that was not used for any of our previous analysis. We used interactions at an average percentile rank >0.9 to define cell-line specific genome-wide maps, which included between 11 696 and 32 308 interactions for each cell line, providing more than 30-fold increase in the number of interactions than detected by 5C.

**Figure 5.**
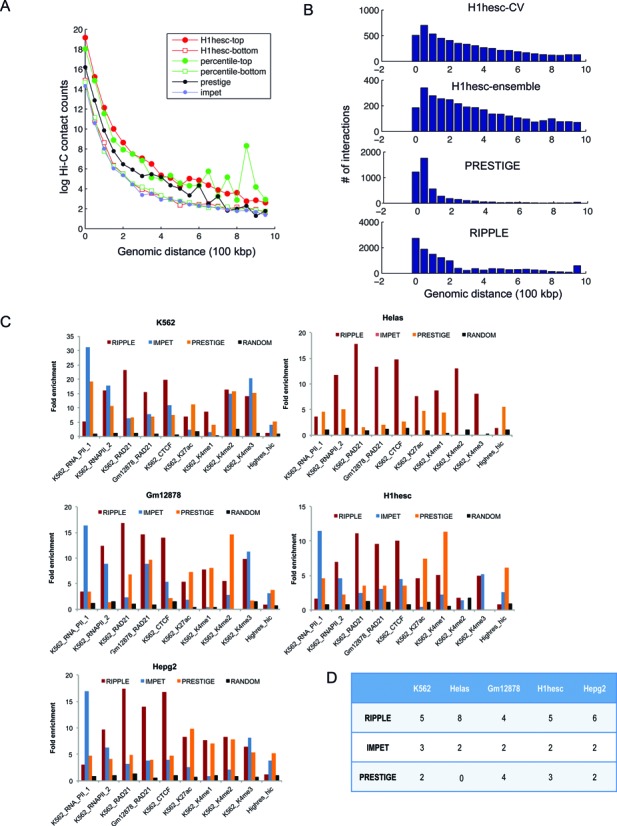
Evaluation of genome-wide enhancer-promoter interaction maps. (**A**) Shown is the distribution of normalized Hi-C contact count frequencies in genome-wide predictions for the H1hesc cell line. H1hesc-top: the interactions in the 90% confidence of the classifier trained using only H1hesc 5C data, H1hesc-bottom: interactions predicted at 10% confidence by the classifier trained only on the H1hesc data, percentile-top and percentile-bottom: Same as in H1hesc-top and bottom but using predictions from the percentile ensemble. PRESTIGE: interactions obtained from the PRESTIGE method, IMPET: interactions obtained from the IM-PET method. (**B**) Distribution of the number of interactions as a function of genomic distance using H1hesc-only classifier (RIPPLE H1hesc CV), Ensemble (RIPPLE H1hesc Ensemble), PRESTIGE and IMPET. (**C**) Fold enrichment of predicted interactions from RIPPLE, IMPET and PRESTIGE in experimental data sets of long-range interactions generated using ChIA-PET or high-resolution Hi-C. Each barplot shows a fold-enrichment measure of the number of recovered interactions of a particular type in the high confidence set of interactions. The RNA_PolII_1 data set is from Li *et al*., whereas the RNA_POLII_2 data set is from Heidari *et al*. All data sets other than Hires_Hi-C are ChIA-PET data sets. (**D**) Shown is the number of data sets for different cell lines (column) in which a method (row) was the best (highest fold enrichment) among the three methods compared. The greater the number the more often was a method ranked the best.

To assess the quality of these genome-wide maps as well as to compare to existing computationally generated interaction maps from two recent methods, IM-PET (30) and PRESTIGE (9), we compared them to available experimental data sets including chromatin capture conformation (Hi-C) and ChIA-PET assays. First, we asked whether the predicted interactions exhibit a significantly high contact count as measured in Hi-C assays for the H1hesc cell line (43). We found that our high confidence interactions (in the 90% percentile) predicted from RIPPLE have significantly higher contact counts compared to low confidence interactions (percentile rank <10%, Figure 5A). Importantly we find that the difference in the high and low confidence interactions in the ensemble based approach is more significant (Paired KS test *P*-value <6E-4) than the interactions predicted by the classified trained only on H1hesc (KS test *P*-value <0.02) demonstrating the advantage of an ensemble based approach. Predictions from IM-PET have a significantly lower contact counts than RIPPLE (KS test *P*-val <0.0013). The margin of contact count difference between PRESTIGE and RIPPLE is not significant (KS test *P*-value<0.15). RIPPLE detected interactions at different distance ranges whereas IM-PET and PRESTIGE had a tendency to detect interactions that were short range (<200 kb, Figure 5B).

We next compared our genome-wide enhancer promoter interactions with those that have been experimentally determined based on ChIA-PET experiments or using high-resolution Hi-C experiment. We had 10 such data sets: high-resolution Hi-C interactions from Jin *et al*. (14), two ChIA-PET data sets, one for PolII and another for CTCF in the K562 cell line (17), and remaining seven ChIA-PET data sets from Heidari *et al*. (18) which included RNA PolII, CTCF, RAD21 and multiple chromatin marks in K562 and Gm12878 cell lines (Figure 5C). Our metric for evaluating these genome-wide maps is fold enrichment, which assesses the fraction of interactions predicted by RIPPLE (or any of the other two computational methods) that overlapped with experimentally detected measurements, compared to the number of interactions expected by random chance (Materials and Methods). We found that RIPPLE-derived interactions were significantly enriched in the ChIA-PET data sets and this enrichment was typically higher than by the other methods. The relatively lower enrichment in the high-resolution Hi-C data sets, compared to other cell lines, is likely due to the fact these these interactions were detected in the IMR90 cell line. Furthermore the percentile ensemble was much better than the cell-line specific classifier (Supplementary Figure S6). To gain an overall assessment of the methods, we asked for each of the data sets, which method gave the best enrichment. Taken over all data sets, we found that RIPPLE's interactions were best enriched in the largest number of data sets across all cell lines (Figure 5D).

### Properties of genome-wide cell line-specific enhancer-promoter interactions

With the genome-wide interactions in hand, we next examined them for various regulatory and network topological properties. Namely, we examined: (i) enrichment of regulatory signals on enhancers and promoters in the high confidence networks, (ii) enhancer-promoter subnetworks, (iii) the extent of cell line-specificity in our genome-wide maps and possible regulatory mechanisms by which cell line-specificity of interactions is established.

#### Enhancers and promoters in genome-wide maps are enriched for known and novel regulatory determinants of long-range interactions

**Figure 6.**
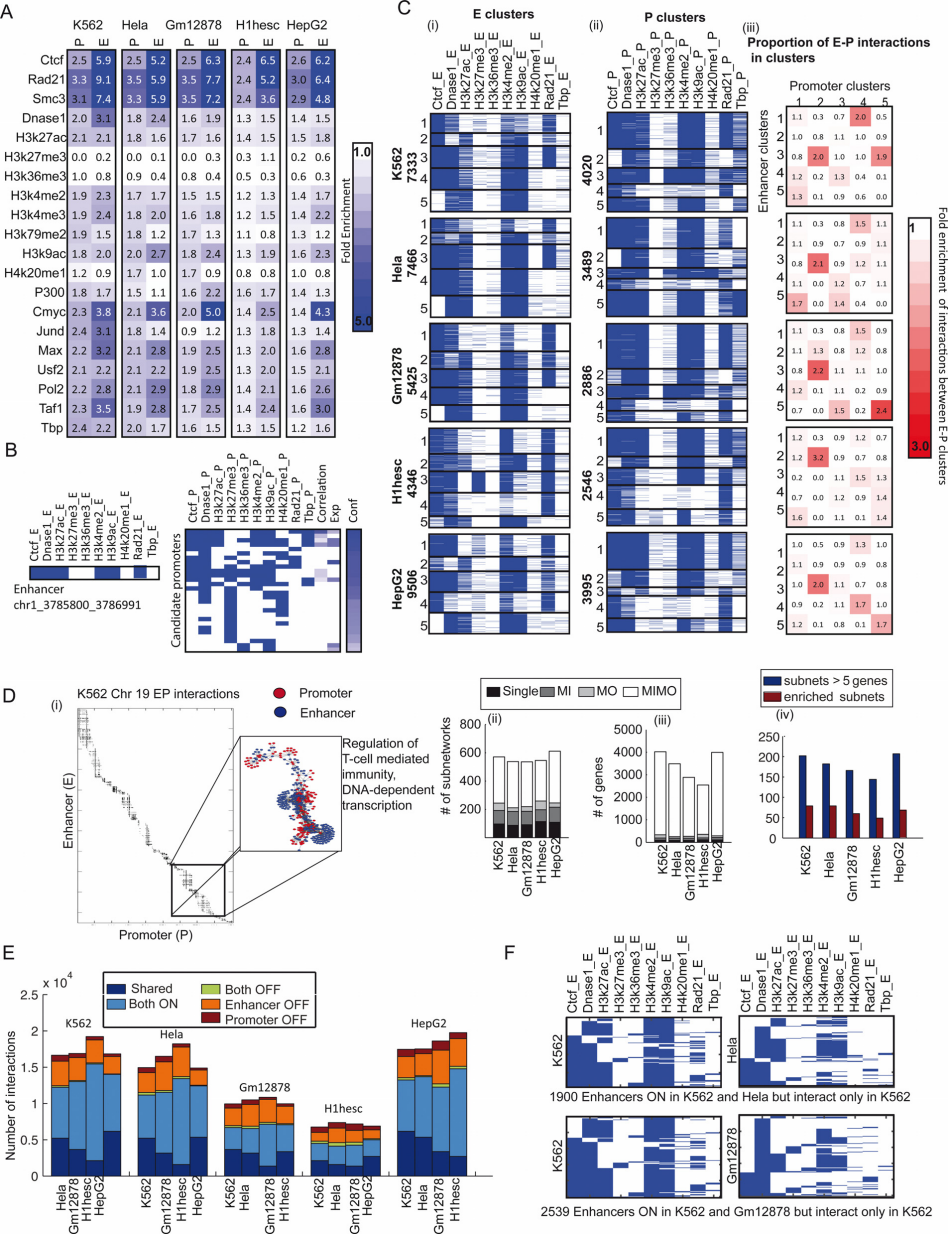
Properties of genome-wide enhancer-promoter interactions. (**A**) Enrichment of various individual genomic signals in the enhancers and promoters in the high confidence networks. The stronger the intensity of blue the better the enrichment. (**B**) Example of an enhancer from the K562 cell line and candidate promoters that are in its 1 MB radius. The promoters are ranked by RIPPLE confidence (Conf; min 0.5). The _E and _P features are binary (0: white, 1: blue), while the Correlation and Expression (Exp) features are continuous. (**C**) Clusters of enhancers and promoters in the five cell lines. (i) Enhancer clusters, each cluster is numbered 1–5, and the number of enhancers are shown on the side. (ii) Promoter clusters, numbered 1–5, with the number of promoters in each cluster shown on the side. Blue indicates the presence of a feature and white indicates absence. (iii) Fold enrichment of interactions between an enhancer cluster (row) to a promoter cluster (column) compared to the expected number of interactions between these clusters. The more red the intensity the greater is the tendency for enhancers from one cluster to interact with promoters from another cluster. (**D**) Enhancer-promoter interaction landscape for Chromosome 19 in K562. (i) The enhancer-promoter interactions for subnetworks extracted from a connected components analysis. The blow-up shows an example set of promoters regulated by multiple enhancers and enriched for transcriptional as well as immune response processes. (ii–iv), Distribution of enhancer-promoter interactions in different types of subnetworks. The majority of the interactions are in the multi-input multi-output subnetworks. Subnetworks are enriched in multiple GO processes, MSigDB gene sets and motifs. (**E**) Proportion of shared and different types of cell line-specific interactions. The same color convention is used as in Figure 3C. Comparison of regulatory signals in enhancers that interact in the K562 cell line but not in Hela (top), and similarly interact in K562 but not in Gm12878 (bottom). The blue color represents the presence of a particular signal in that region. The rows are sorted based on the entries in the first column (CTCF) followed by the second columns, etc (using MATLAB's sortrows function). Rows of each column maintain the ordering of the preceding columns.

The high confidence interactions analyzed in Figure 5 connected between 6105 and 11 313 enhancers regions to 2275 and 6509 genes (Table 2). We asked if the enhancers and promoters in the high confidence network are associated with specific regulatory signals. We find that the enhancers are highly enriched for the three architectural proteins, CTCF, RAD21 and SMC3 (Figure 6A). CTCF and RAD21 enrichments are consistent with the importance of these features in our predictive framework. SMC3 is involved in structural maintenance of chromosomes and is part of the cohesin complex, which includes RAD21. Both CTCF and cohesin (RAD21 and SMC3) are known to be important for maintaining chromosomal loops that can link enhancers to their target genes (22). An example of interactions mediated via the presence of RAD21 and CTCF is shown in Figure 6B. In addition to these architectural proteins, the enhancers were enriched for activating marks of transcription such as H3K4me2, H3K4me3, H3K9ac and H3K27ac. The enrichment of architectural proteins and activating marks in our interacting enhancers and promoters provide external support for our predictions and suggest that these enhancers are likely regulating their predicted target genes. We also found enrichment of RNA PolII and general transcription factors, namely CMYC, MAX and TAF (TBP associated factors) at the enhancers. RNA PolII is a hallmark of transcription factories, defined as large nuclear compartments in which multiple genes are coordinately transcribed, and are important components of the three-dimensional organization of the genome (47). Among the different transcription factors, the enrichment of CMYC was most striking, coming close to CTCF and cohesin. CMYC is a helix-loop-helix zipper transcription factor and an oncogene with roles in diverse processes including proliferation, cell cycle and apoptosis (48). CMYC is considered an architectural protein that disrupts chromatin organization (49), and may influence chromatin state through up-regulation of a histone acetyltransferase (48,49). Interestingly CMYC's enrichment was weakest in the H1hesc cell line, but similar across the differentiated cell lines implicating CMYC in establishing differences in chromosome organization between differentiated and undifferentiated cell types. The MAX transcription factor forms a heterodimer with CMYC to drive gene expression of specific gene regulatory modules (50) and could also be associated with the formation of transcription factories. Finally, TAF has been shown to co-bind with CTCF and cohesin components to establish chromosomal loops during embryonic stem cell differentiation (51). Taken together our enrichment results identify known regulatory factors such as CTCF and cohesin (RAD21, SMC3), and predict candidate general transcription factors (e.g. CMYC) that might be important for establishing 3D genome organization, especially in differentiated cells.

**Table 2.** Number of enhancer-promoter interactions in the high confidence (90% percentile) genome-wide networks, as well as the total number of input enhancers and promoters available in each cell line.

Cell line	EP Interactions	Enhancers (E)	Promoters (P)	Total Enhancers	Total Promoters
K562	28 428	7333	4020	118 866	32 349
Hela	25 283	7466	3489	110 415	30 338
Gm12878	16 343	5425	2886	104 159	28 001
H1hesc	11 696	4346	2546	81 618	26 233
HepG2	32 308	9506	3995	115 128	30 277

#### Combinations of architectural proteins and chromatin marks discriminates between different classes of interacting enhancers

Given that the enhancers and promoters in our high confidence interactions are enriched for individual regulatory signals, we asked if combinations of different regulatory signals can define different classes of interacting enhancers and promoters. We applied K-means clustering to these enhancers and promoters to identify five enhancer and five promoter clusters in each cell line. We mapped the observed pattern of signals in a cluster to a cluster ID from 1 to 5 such that the same cluster ID would represent the same pattern across cell lines as much as possible (Table 3). Thus cluster *i* in one cell line would have as close as possible a pattern as cluster *i* in another cell line. The majority of the patterns in a cluster were observed in other cell lines (Table 3). We repeated the procedure for both the enhancer and promoter clusters.

**Table 3.** Different clusters of enhancers and promoters in high confidence (90% percentile) interaction networks

ClusterID	Regulatory Signals	Comments
Enhancers		
1	CTCF, DNase I, H3K4me2, RAD21	Weak in K562
2	CTCF, H3K27ac, H3K4me2, H3K9ac, RAD21	Weak in Gm12878
3	CTCF, DNase I, H3K4me2, H3K9ac, RAD21	H3K9ac missing in H1hesc
4	CTCF, DNase I, H3K4me2, H3K9ac	RAD21 present, H3K9ac missing in H1hesc, HepG2
5	DNase I, H3K27ac, H3K9ac, H3K4me2	
Promoters		
1	CTCF, DNase I, H3K27ac, H3K4me2, H3K9ac, RAD21, TBP	No TBP in Hela, and weak in Gm12878
2	DNase I, H3K27ac, H3K4me2, H3K9ac, TBP	
3	CTCF, DNase I, H3K27ac, H3K36me3, H3K4me2, RAD21	Has TBP in K562
4	CTCF, DNase I, RAD21	DNase I weakly present in K562
5	CTCF, DNase I, H3K4me2, RAD21	RAD21 absent in K562, TBP, H3K27ac, H3K9ac present in K562 and Hela

The enhancer clusters (ECs), were primarily defined by combinations of CTCF, DNase I, H3K27ac, H3K9ac and RAD21 (Figure 6C, (i)). CTCF was present in all clusters in all cell lines except for EC5, which represents a unique set of enhancers that are not dependent upon CTCF for interactions. EC3 in H1hesc represented another set of enhancers being the only group that is associated with H3K27me3, a mark associated with repressed expression. The promoter clusters (PCs) were defined additionally by the presence or absence of H3K36me3, TBP and H4K20me1 (Figure 6C, (ii)). PC3 of K562, Gm12878 and Hela was associated with H3K36me3. H4K20me1 genome-wide showed a weaker association with specific clusters, most notably with PC3 of Hela. These results suggest that H3K36me3 and H4K20me1 tend to be important features associated with promoters and likely mark active genes for transcription. This is consistent with the roles of these marks in transcriptional elongation (H3K36me3). H4K20me1 has been shown to be involved in both gene activation and repression, but is promoter proximal (52).

We next asked whether the enhancers in each cluster had a tendency to interact with promoters in another cluster (Figure 6C, (iii)). In all but the H1hesc cell line, we found that enhancers from EC4 interacted with genes in PC2 and were associated with amine metabolism. We also found that enhancers from EC1 significantly interacted with promoters from PC4, and genes in PC4 are associated with RNA metabolism and transcriptional processes (Supplementary Figure S7). Taken together these clusters specify, at a coarse level, different types enhancers and promoters, each type defined by combinations of chromatin marks and regulatory proteins associated with them, and enhancers of some types exhibited a preference to interact with promoters of another type. Such types and interaction preferences were observed across multiple cell lines suggesting these are general properties shared across multiple cell lines.

#### Enhancer-promoter interactions are organized into subnetworks that are enriched in distinct biological processes

To gain a more fine-grained understanding of the interaction patterns, we computed for every enhancer region the number of genes it is predicted to connect (*out degree*), and for every gene, the number of enhancers that are predicted to be connected to it (*in degree*). On average an enhancer was predicted to regulate 3–4 target genes, while a gene was predicted to be regulated by 10–21 enhancers suggesting that enhancers and promoters form combinatorial interaction patterns with sets of enhancers regulating sets of promoters (Figure 6D). To determine the connectivity patterns among enhancers and promoters, we identified connected components in the interaction network of enhancers and promoters. Enhancers and promoters can interact in the four possible ways: (i) Single interactions (Single) involving an interaction between one enhancer and one promoter, (ii) Multi-input (MI) components, involving a promoter predicted to be regulated by multiple enhancers, (iii) Multi-output (MO) components, involving an enhancer regulating multiple genes, (iv) Multi-input Multi-output (MIMO) components, involving multiple enhancers predicted to regulate multiple genes. We found that most of the interactions belonged to connected components of the MIMO class (Figure 6D (ii)), and accounted for 85–92% of the genes. Furthermore, several of the MIMO and MO graph components included large numbers of genes (e.g. >50 genes). Such connected components represent potentially coordinately regulated sets of genes. Of the subnetworks that included five or more genes, 32–43% were enriched in either a Gene Ontology process, a regulatory element from the MSigDB database, or a KEGG or REACTOME pathway (Figure 6D (iii), Supplementary Figure S8). For example, in K562, we found a large connected component on chromosome 19, composed of 145 genes (Figure 6D (i)) that was enriched in transcriptional processes (DNA-dependent transcription) as well as innate immunity (regulation of leukocyte/lymphocyte medicated immunity and cytotoxicity). The processes enriched in these subnetworks included both shared and cell line-specific processes. For example, we found several subnetworks that were associated with house keeping functions such as RNA metabolism, DNA-dependent transcription to be enriched in all cell lines, while lipid biosynthesis was unique to HepG2, and cell morphogenesis to H1hesc. The enrichment of distinct biological processes, *cis*-regulatory elements and curated pathways in these gene sets are indicative of coordinated regulatory units and reveal a complex network of interaction patterns that is organized into smaller sub-units of multi-input multi-output components.

#### Combination of architectural protein binding and chromatin state is important for an enhancer to interact

We classified the high confidence interactions between any pair of cell lines as shared or cell-line specific, where an interaction was called specific to cell line A if its probability of interaction was in the 90% percentile in cell line A, but below the 80% percentile in cell line B. We classified cell-line specific interactions into the four categories described in Figure 3A using the same criteria of ON as defined before: (i) Both OFF, (ii) enhancer-OFF promoter-ON, (iii) enhancer-ON promoter-OFF, (iv) Both-ON. As in the 5C case, we found that the Both-ON and enhancer-OFF categories were the two dominating categories of cell-line specific interactions (Figure 6E). We investigated the enhancers of the Both-ON cell line-specific interactions (interaction observed in cell line A but not in B) to determine whether such enhancers have lost some critical regulatory signals that make it conducive for a long range interaction. Specifically for every pair of cell lines we obtained those enhancers that were ON in both cell lines, but interacted in one cell line but not in the other. Considering K562 as an example of cell line A and Hela as an example of cell line B, we found that such enhancers tend to be associated with CTCF, and some times with H3K27ac if not CTCF, along with DNase I, in K562, while in Hela they are depleted for at least one of the important signals, most notably CTCF (Figure 6F). Interesting, in both cell lines enhancers remained associated with DNase I suggesting that the individual indicators of active chromatin are not sufficient for an enhancer to interact with its target gene. We observe a similar trend between K562 and Gm12878, and more broadly across other pairs of cell lines (Supplementary Figure S9), where the enhancers that do not interact seem to have lost either CTCF or H3K27ac. To summarize, our analysis of different types of cell line-specific interactions exhibit similar distributions of different types of cell line-specific interactions as in 5C, and further suggests that a combination of architectural protein binding and chromatin state is important for establishing an enhancer's propensity to interact with target genes.

## DISCUSSION

A central challenge in mammalian tissue-specific gene expression patterns is to understand the mechanisms by which enhancers interact and regulate their target genes that are not necessarily close in linear space (4,53). While methods based on chromosome capture conformation are maturing to detect interactions among two DNA sequence elements (54), identifying cell line-specific on a genome-scale and examining the role of different one-dimensional regulatory signals for establishing these interactions is still a challenge. To address this challenge, we have developed RIPPLE, a computational approach to predict cell line-specific long range interactions between enhancers and promoters on a genome-wide scale in multiple cell lines, and analyzed these interactions to identify both known and novel determinants of long-range gene regulation.

An important aspect of our approach is that it started with a large collection of regulatory genomics data sets and used a predictive framework to identify important data sets for computationally predicting enhancer-promoter interactions. Our predictive learning framework combined the strengths of two machine learning approaches: the predictive power of Random Forests and structured sparsity based multi-task learning to determine important data sets for multiple cell lines. Our predictive framework enabled us to study known players of long-range gene regulation (e.g. CTCF and cohesin) together with additional components of the transcription machinery (e.g. chromatin marks, general transcription factors), many of which have not been thoroughly characterized in the context of enhancer-promoter interactions. This was important to study the contribution of one-dimensional signals to three-dimensional organization of the genome, in which enhancer-promoter interactions are critically important.

Our feature analysis identified that architectural proteins, activating histone modification marks and a general transcription factor (e.g. TBP) are important for establishing these interactions. The importance of a general transcription factor in establishing interactions was a surprising discovery, but is consistent with the interplay of CTCF and cohesin components with transcription factors to trigger cell-type specific gene expression programs (51). The presence of RNA PolII, CMYC and TAF1 can also be due to the formation of transcription factories, another important mechanism of long-range gene regulation (47). We used TBP in our predictions, however, we believe that the other TFs such as JUND, CMYC or USF1 could as well be used.

Among the marks that we found as important for long range regulatory interactions were known marks such as H3K9ac, H3K7ac and H3K4me2, as well as marks that have thus far not been associated with enhancer-promoter interactions, namely H3K36me3 and H4K20me1. H4K20me1 has been associated with gene repression as well as activation (52). More recent genome-wide ChIP-seq studies have shown that the H4K20me1 is associated with active transcription of genes. H3K36me3 is known to be associated with transcriptional elongation canonically, however recent studies suggest that H3K36me3 is found on active enhancers indicative of a transcriptionally active chromatin state (55). H3K36me3 together with other marks was also shown to be enriched in regions that interact with promoter regions as detected using Hi-C capture studies (15). The addition of H3K27me3 was surprising, however, it was voted by both the Random Forests and Group Lasso and is likely associated with poised enhancers (46). H3K27me3 was also observed to have enrichment in H1hesc, which is consistent with the occurrence of such enhancers typically in ES cells (46). Future investigation of the impact of perturbing such marks on the long-range interaction landscape will be informative for gaining a deeper understanding of the principles governing such interactions.

Our cross-cell line prediction shows that a classifier trained on one cell line can predict interactions in other cell lines. However the accuracy of classifiers can vary from one test cell line to another, making the choice of the classifier non-trivial. Ensemble approaches that aggregate predictions from multiple classifiers are a natural way to approach this problem. We examined three different ways of combining predictions from multiple cell lines and found that the overall performance of different ensembles was similar. We used the percentile rankings of predictions which has the advantage of being easily extensible to classifiers trained on more cell lines. While the ensemble predictor was close to the best performing cell line more systematic approaches to combine shared information between different cell lines, (e.g. by considering different weighting strategies of different cell lines) will be an important direction of future work.

Our analysis of both small-scale 5C and our genome-wide predictions showed that cell line-specific interactions are of two major categories. In one, cell line specificity of interactions is mediated by the loss of activity of an enhancer, and in another, cell line-specificity of interactions can occur even if the enhancer is active (as determined by the presence of DNase I, H3K27ac or H3K9ac mark). We found such enhancers tend to have a subset of the regulatory signals needed to establish an interaction. For an enhancer to interact it must have a combination of activating marks, and binding by one or both of the architectural proteins. CTCF was by far the most important determinant of these cell line-specific interactions.

We analyzed our global maps to characterize the network properties of interactions among enhancers and promoters. At a coarse scale we identified different classes of interacting enhancers and promoters, that were discriminated by the presence of different combinations of chromatin marks and architectural proteins. Promoter clusters were additionally associated with elongation specific marks namely H3K36me3 and H4K20me1. A finer scale analysis identified connected subnetworks of enhancers and promoters forming multi-input multi-output subnetworks that were significantly enriched for Gene Ontology biological processes.

In conclusion, we have developed a systematic framework to predict interactions between enhancer and promoter regions across multiple cell lines by integrating 3C data sets with one-dimensional regulatory signals measured in chromatin marks and TF ChIP-seq data sets. Our approach identified that a combination of architectural proteins, transcription factors and histone modifications are needed for establishing long-range regulation. Our approach can be easily applied to new cell lines to predict interactions among new genomic loci in the same cell line, or be used to generalize to new cell lines where we do not have any training data. We provide our genome wide interactions as a web-based resource (http://pages.discovery.wisc.edu/∼sroy/ripple/queryg.php) that users can query using their regions of interest. Our associated predictions will serve as a useful resource to query potential interacting partners for a new gene or locus of interest and prioritize enhancers for follow up studies using targeted validation.

## SUPPLEMENTARY DATA

Supplementary Data are available at NAR Online.

SUPPLEMENTARY DATA
