# A predictive modeling approach for cell line-specific long-range regulatory interactions

**DOI:** 10.1093/nar/gkv1181

**Published:** 2015-11-05

**Authors:** Sushmita Roy, Alireza Fotuhi Siahpirani, Deborah Chasman, Sara Knaack, Ferhat Ay, Ron Stewart, Michael Wilson, Rupa Sridharan

**Affiliations:** 1 Department of Biostatistics and Medical Informatics, University of Wisconsin-Madison, Madison, WI, USA; 2 Wisconsin Institute for Discovery, 330 N. Orchard Street, Madison, WI, USA; 3 Department of Computer Sciences, University of Wisconsin-Madison, Madison, WI, USA; 4 Department of Genome Sciences, University of Washington, Seattle, WA, USA; 5 Morgridge Institute for Research, Madison, WI 53715, USA; 6 Genetics & Genome Biology Program, Hospital for Sick Children (SickKids) and Department of Molecular Genetics, University of Toronto,Toronto, ON, Canada; 7 Department of Molecular Genetics, University of Toronto, ON, Canada; 8 Department of Cell and Regenerative biology, University of Wisconsin, Madison, WI 53715, USA

*Nucl. Acids Res*. 43 (18): 8694–8712. doi: 10.1093/nar/gkv865

The authors wish to make the following corrections to Figure 5:

**Figure 5.**
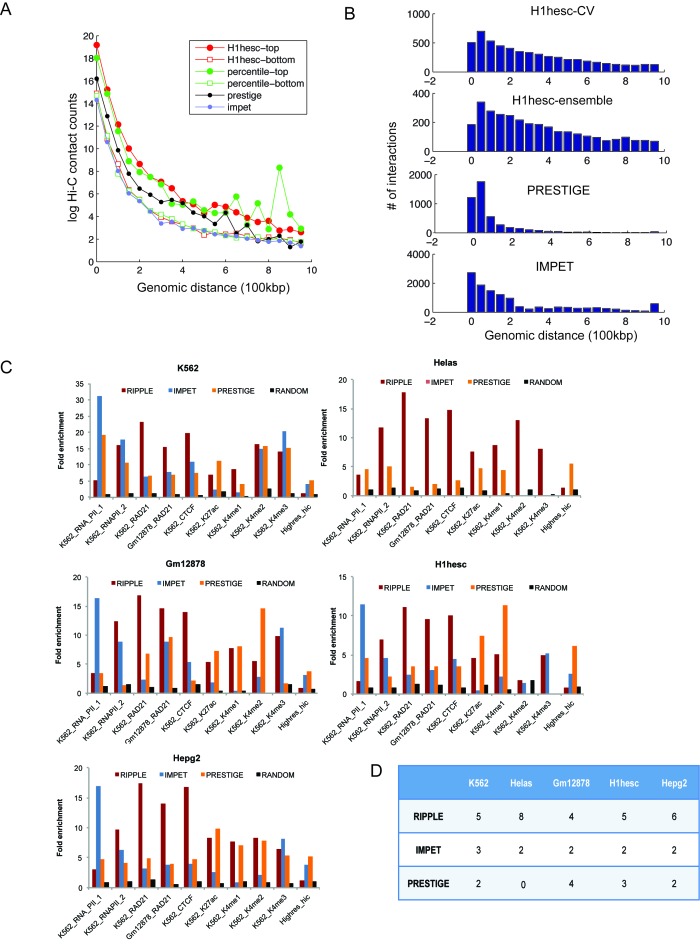
Evaluation of genome-wide enhancer-promoter interaction maps. (**A**) Shown is the distribution of normalized Hi-C contact count frequencies in genome-wide predictions for the H1hesc cell line. H1hesc-top: the interactions in the 90% confidence of the classifier trained using only H1hesc 5C data, H1hesc-bottom: interactions predicted at 10% confidence by the classifier trained only on the H1hesc data, percentile-top and percentile-bottom: Same as in H1hesc-top and bottom but using predictions from the percentile ensemble. PRESTIGE: interactions obtained from the PRESTIGE method, IMPET: interactions obtained from the IM-PET method. (**B**) Distribution of the number of interactions as a function of genomic distance using H1hesc-only classifier (RIPPLE H1hesc CV), Ensemble (RIPPLE H1hesc Ensemble), PRESTIGE and IMPET. (**C**) Fold enrichment of predicted interactions from RIPPLE, IMPET and PRESTIGE in experimental data sets of long-range interactions generated using ChIA-PET or high-resolution Hi-C. Each barplot shows a fold-enrichment measure of the number of recovered interactions of a particular type in the high confidence set of interactions. The RNA_PolII_1 data set is from Li et al., whereas the RNA_POLII_2 data set is from Heidari et al. All data sets other than Hires_Hi-C are ChIA-PET data sets. (**D**) Shown is the number of data sets for different cell lines (column) in which a method (row) was the best (highest fold enrichment) among the three methods compared. The greater the number the more often was a method ranked the best.

In Figure 5B, the bottom panel is incorrectly labelled as RIPPLE. The correct method and label, as stated in the figure caption, should be IMPET. A corrected Figure is provided below.

The results and conclusion of the article are not affected and remain valid. The authors apologise to the readers for the inconvenience caused.

