# Supplementary material for: A predictive modeling approach for cell line-specific long-range regulatory interactions
Source: Nucleic Acids Res. 2015 Oct 10;43(18):8694–712. doi: 10.1093/nar/gkv865 (PMC4605315; doi:10.1093/nar/gkv865)
Supplement: SUPPLEMENTARY DATA [file supp_43_18_8694__index.html]

A predictive modeling approach for cell line-specific long-range regulatory interactions — SUPPLEMENTARY DATA 

# A predictive modeling approach for cell line-specific long-range regulatory interactions

## SUPPLEMENTARY DATA

- SUPPLEMENTARY DATA
