# Supplementary material for: A predictive modeling approach for cell line-specific long-range regulatory interactions
Source: Nucleic Acids Res. 2015 Oct 10;43(18):8694–712. doi: 10.1093/nar/gkv865 (PMC4605315; doi:10.1093/nar/gkv865)
Supplement: SUPPLEMENTARY DATA [file supp_gkv865_nar-01523-n-2015-File002.pdf]

Supplementary Figures and Legends

Supplementary Figure 1

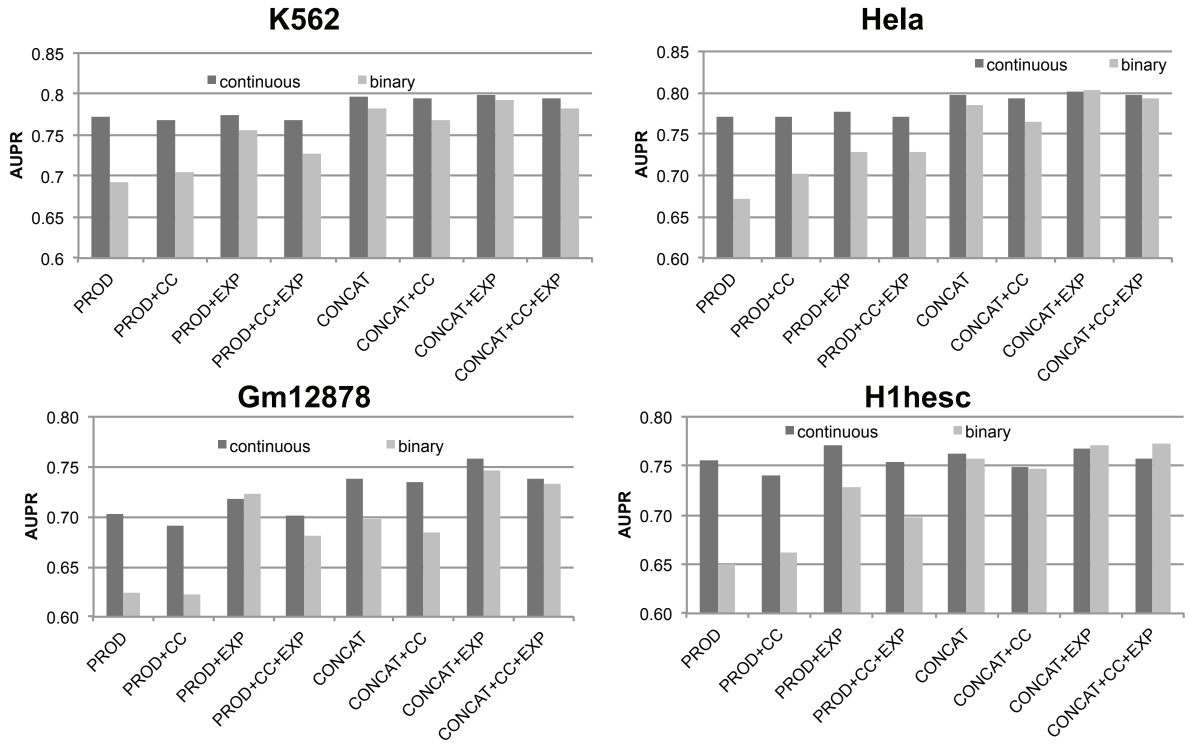

Supplementary Figure 1. Comparison of different feature encodings. Area under precision-recall curve (AUPR) values for different enhancer-promoter interaction feature representations (CONCAT and PROD) for four cell lines: K562, Hela S3 (Hela), Gm12878 and H1hesc using a Random Forests classifier. The higher the AUPR, the better the feature encoding. PRODUCT is generally worse than CONCAT, and binary and continuous are similar for CONCAT. CC: Correlation, EXP: Expression levels.

Supplementary Figure 2

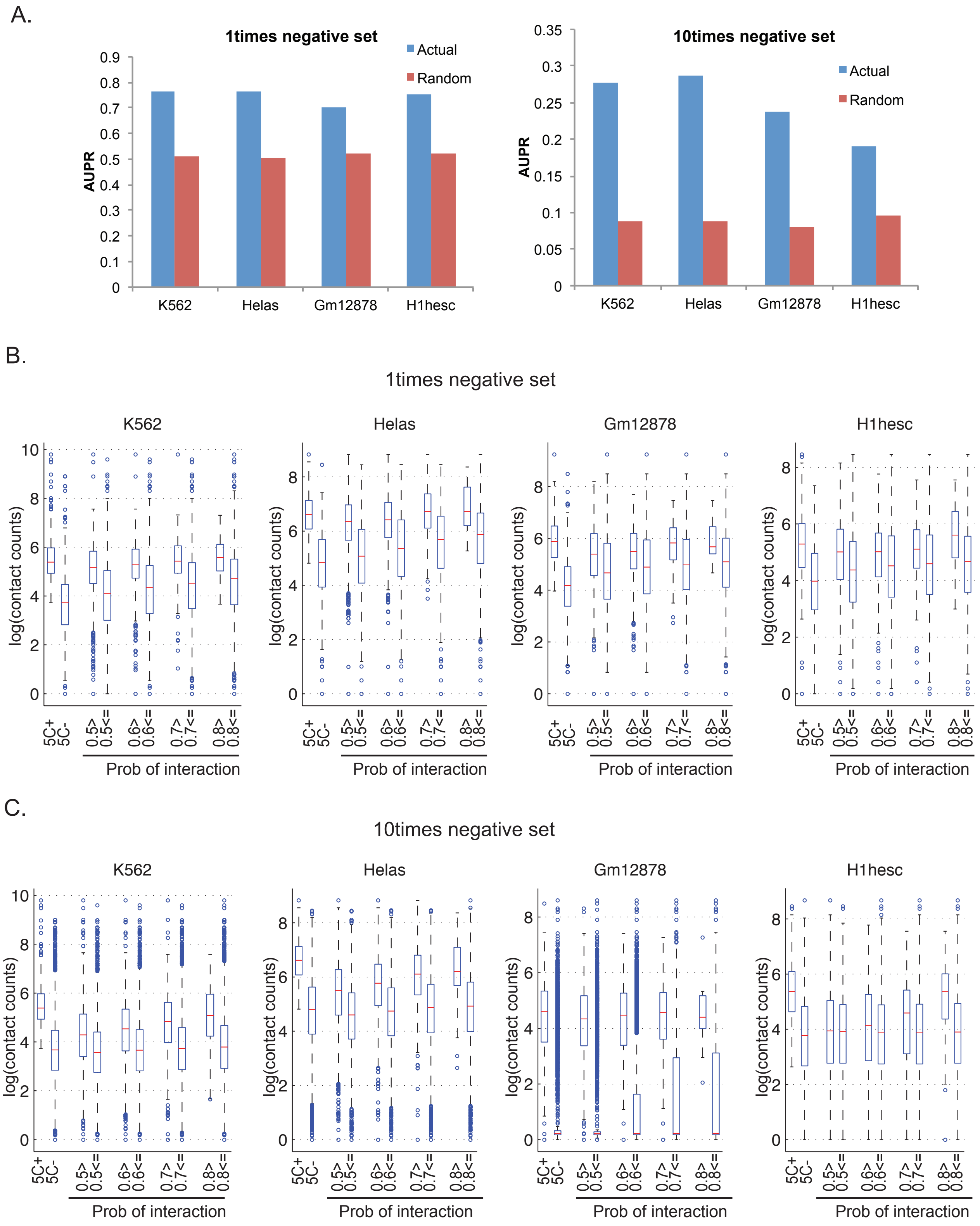

Supplementary Figure 2. Effect of different negative set size on classification performance. A. Shown are the AUPRs for classifiers on a negative set of size equal to (1times) and greater than (10 times) the size of the positive set. The blue bar corresponds to RANDOM, which is obtained by shuffling the confidence values associated with each pair. **B.** Differences in 5C contact counts in true interactions (5C+) and true non-interactions (5C-), as well as in predicted interactions and non-interactions at different classification probabilities (0.5, 0.6, 0.7, 0.8). The left set of bar plots is for the negative set size equal to the size of the positive set (1times), while the right set of bar plots is for the negative set size 10 times larger than the size of the positive set (10times). In both negative set sizes, we observe a significant different in predicted interacting and non-interacting pairs which is comparable to what is observed in 5C.

Supplementary Figure 3

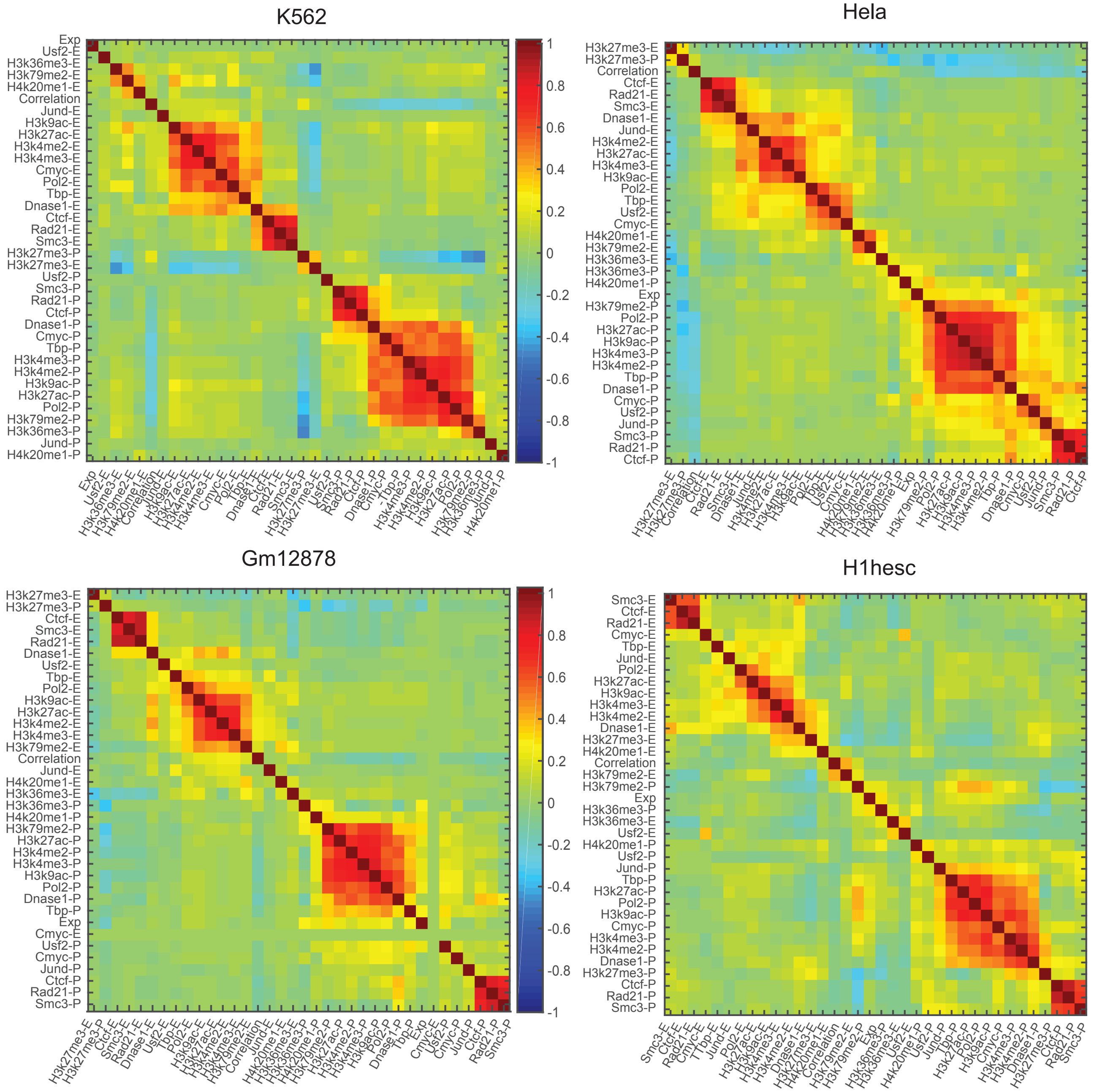

Supplementary Figure 3. Correlation of various datasets across the four cell lines. Correlations were computed for each feature (E: enhancer, P: promoter) in each cell line. Rows and columns were ordered based on optimal leaf ordering of hierarchical clustering of the features using 1-Pearson's correlation as the distance metric.

Supplementary Figure 4

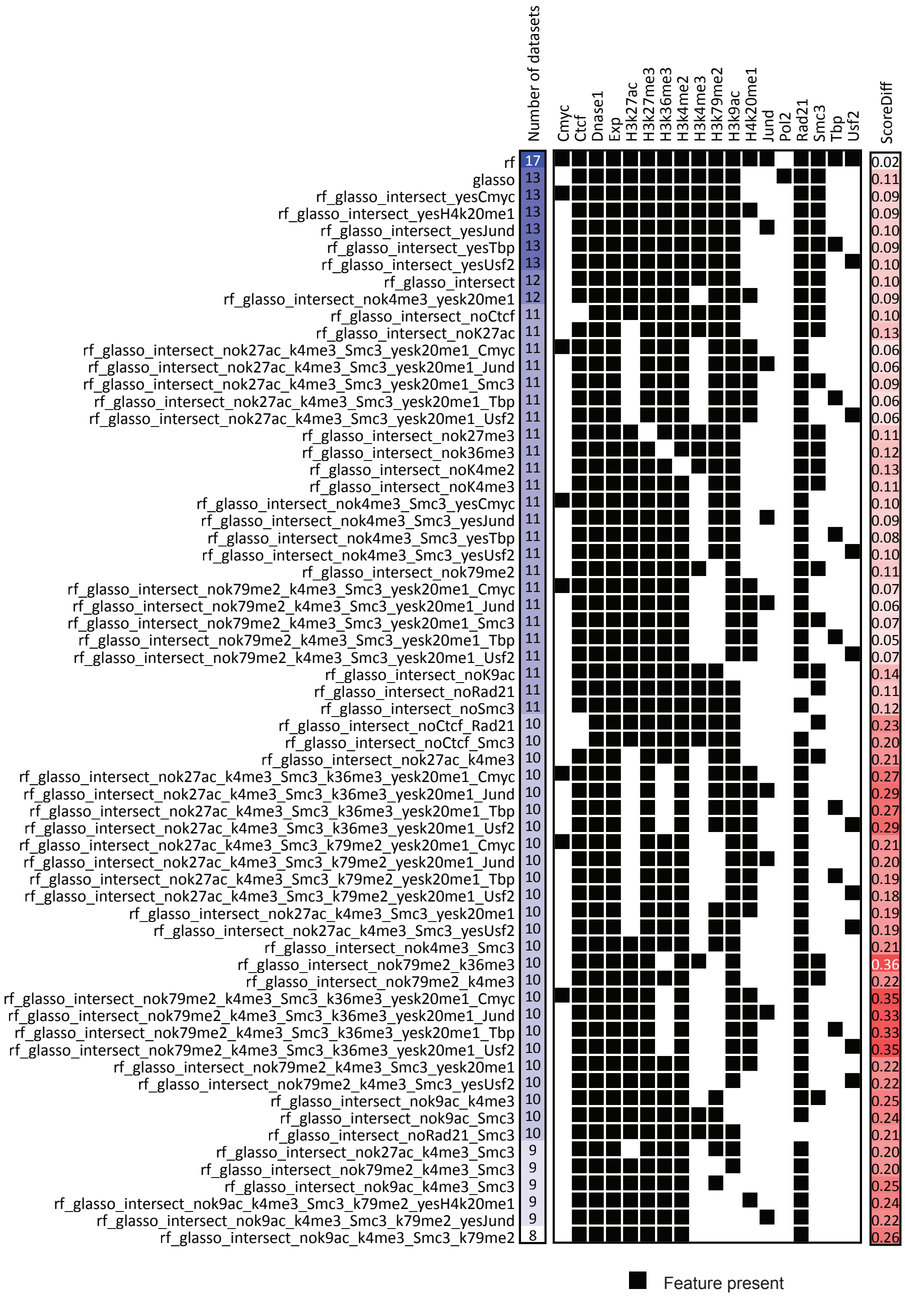

Supplementary Figure 4. Combinations of features tested. Each row represents a particular feature combination tested. The ScoreDiff shows the difference in performance compared to a Random Forests classifier trained on all 23 datasets summed over all four cell lines. *rf* represents the 17 datasets selected by Random Forests (RF) feature selection. *glasso* shows the datasets selected using Group Lasso. *rf_glasso_intersect* represents the features using the intersection of Group Lasso and RF-selected features. All other feature combinations represent refinements of this feature set.

Supplementary Figure 5

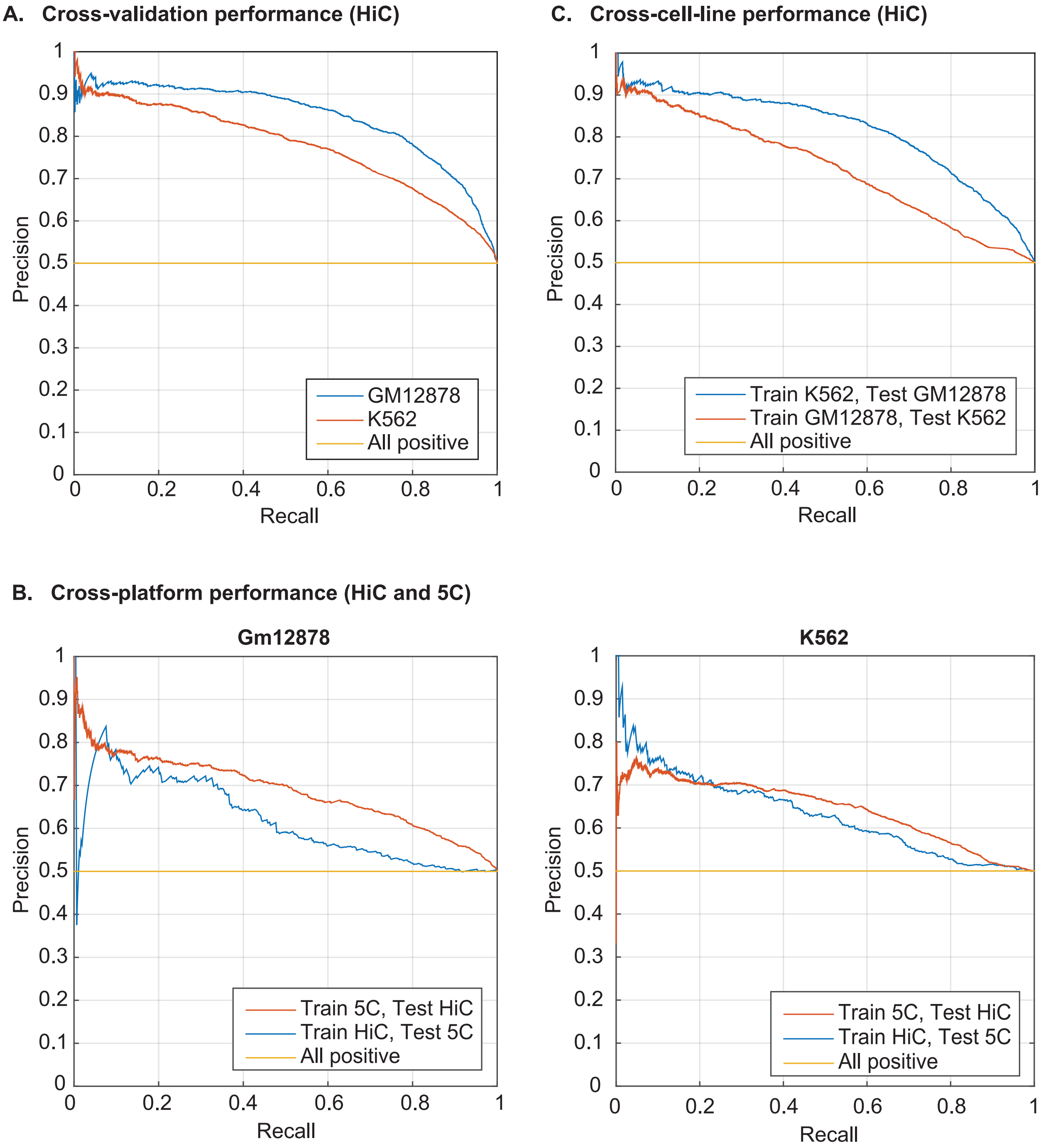

Supplementary Figure 5. RIPPLE generalizes to HiC data. Each panel is a precision-recall curve. The yellow horizontal line, labeled "All positive", represents the precision obtained by classifying all candidate pairs as positive interactions. **A.** Shown are precision-recall curves generated from 10-fold cross-validation of a Random Forests classifier trained with the features used in RIPPLE (11 datasets) for Hi-C distal-promoter interactions from two cell lines, Gm12878 (blue) and K562 (red). **B.** Precision-recall curves demonstrating the performance of the Random Forests classifier trained on interactions from one platform (Hi-C or 5C) and tested on the other. Each plot shows results for a different cell line, Gm12878 and K562. **C.** Performance of Random Forests classifier trained on Hi-C interactions for one cell line and tested on another (between Gm12878 and K562).

Supplementary Figure 6

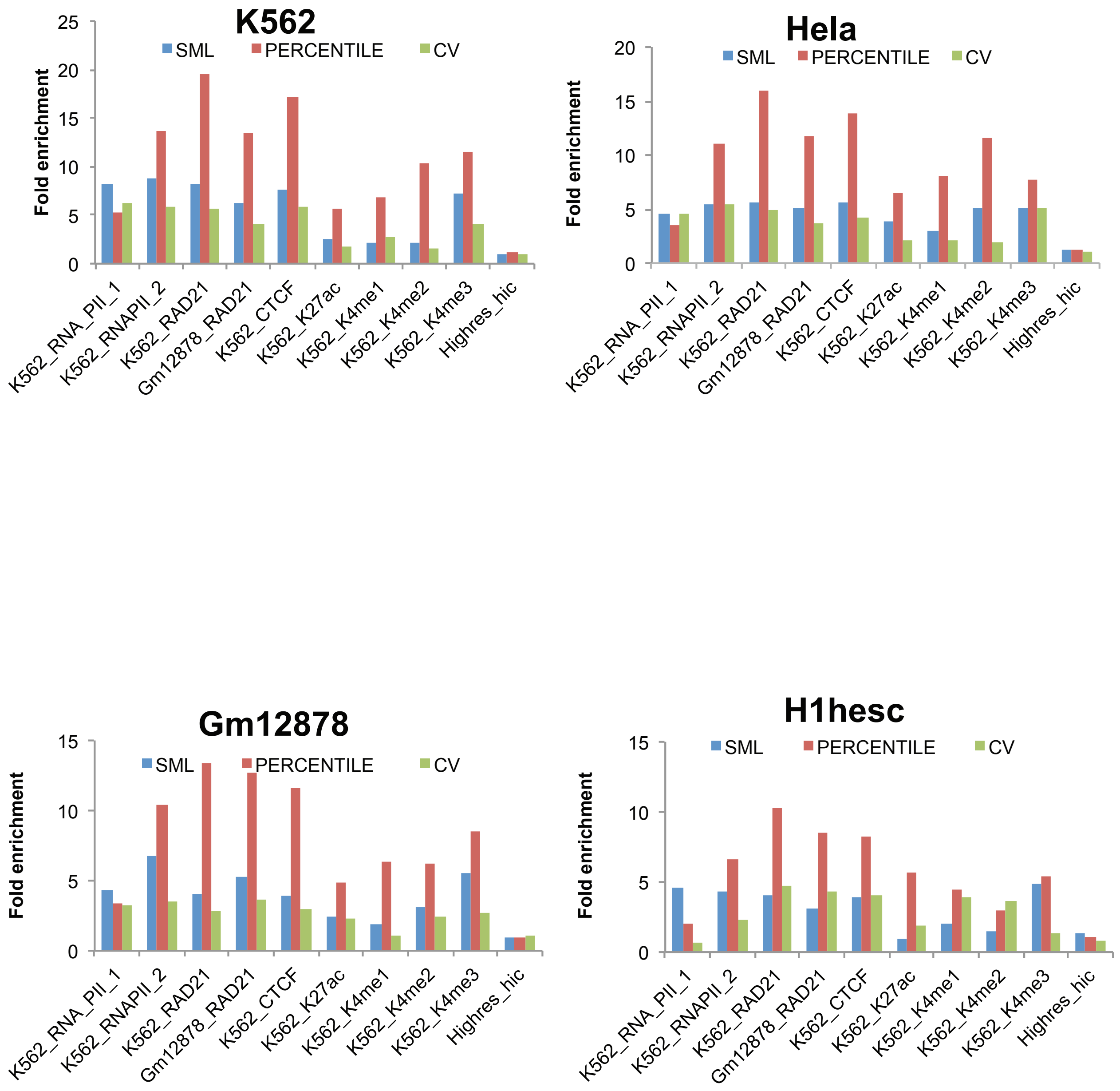

Supplementary Figure 6. Comparison of genome-wide predictions and ensemble classifiers. Shown are the fold enrichments of different ChIA-PET and high resolution Hi-C datasets in the genome-wide networks derived using cell-line specific classifier (CV) and the two ensemble approaches, PERCENTILE and SML. Each bar plot is associated with a cell line. HepG2 is not shown because we did not have a classifier trained on this.

Supplementary Figure 7

Gene Ontology Processes

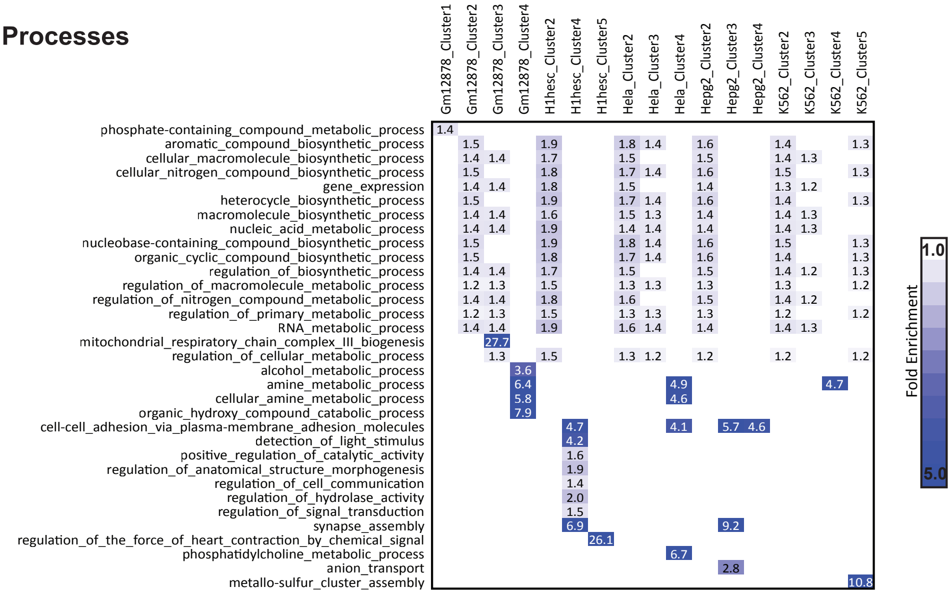

KEGG/REACTOME/BIOCARTA Pathways

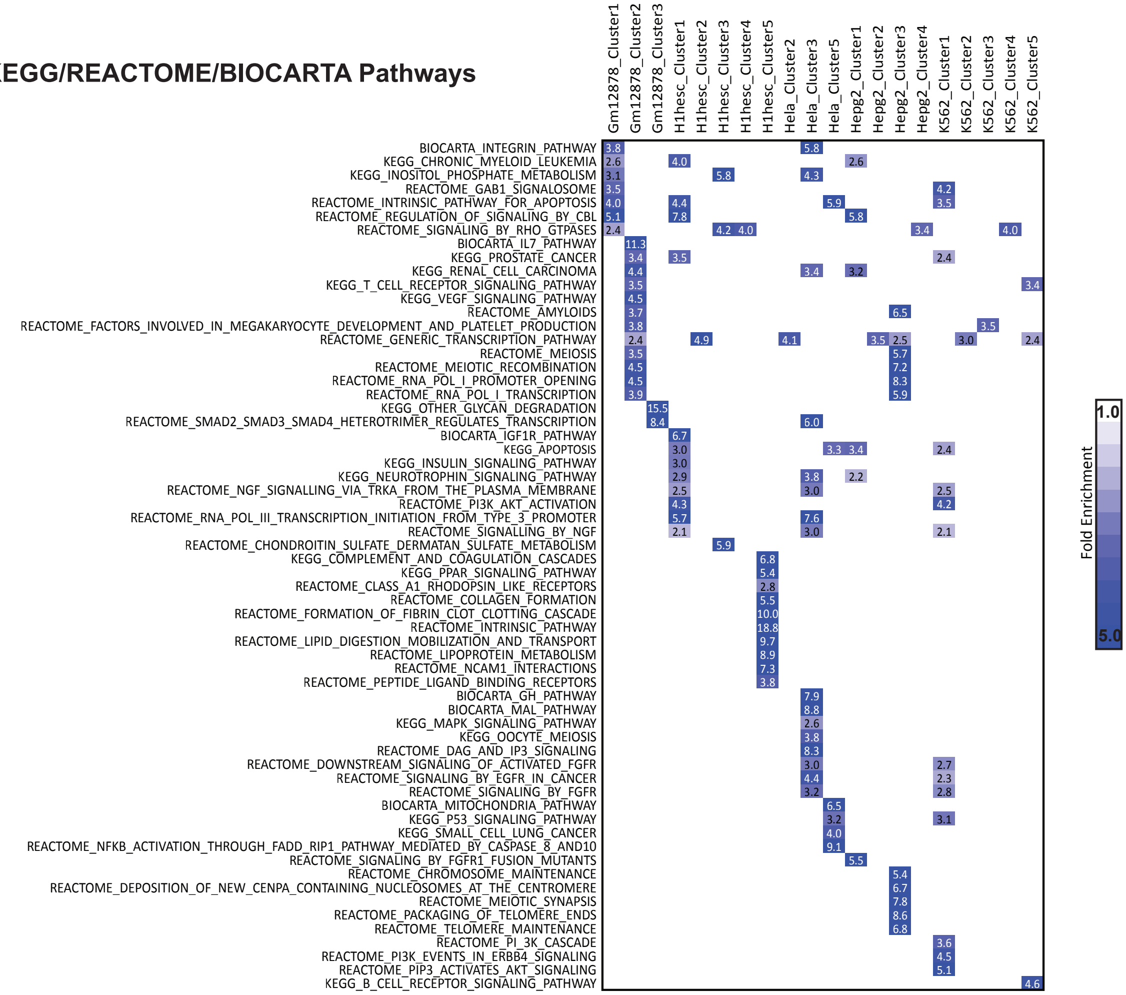

Supplementary Figure 7. Gene Ontology (GO) and Pathway enrichment of promoter clusters Shown are the GO and curated pathways from MSigDB, which includes KEGG, BioCARTA and REACTOME pathways in five promoter clusters for each of the five cell lines. Clusters that did not have any enrichment are not shown. The blue intensity corresponds to the fold enrichment of the process in the specific cluster. Terms were selected such that they were among the top 5 significant terms (FDR corrected Hypergeometric P-value <0.05) in at least one of the cell lines.

Supplementary Figure 8

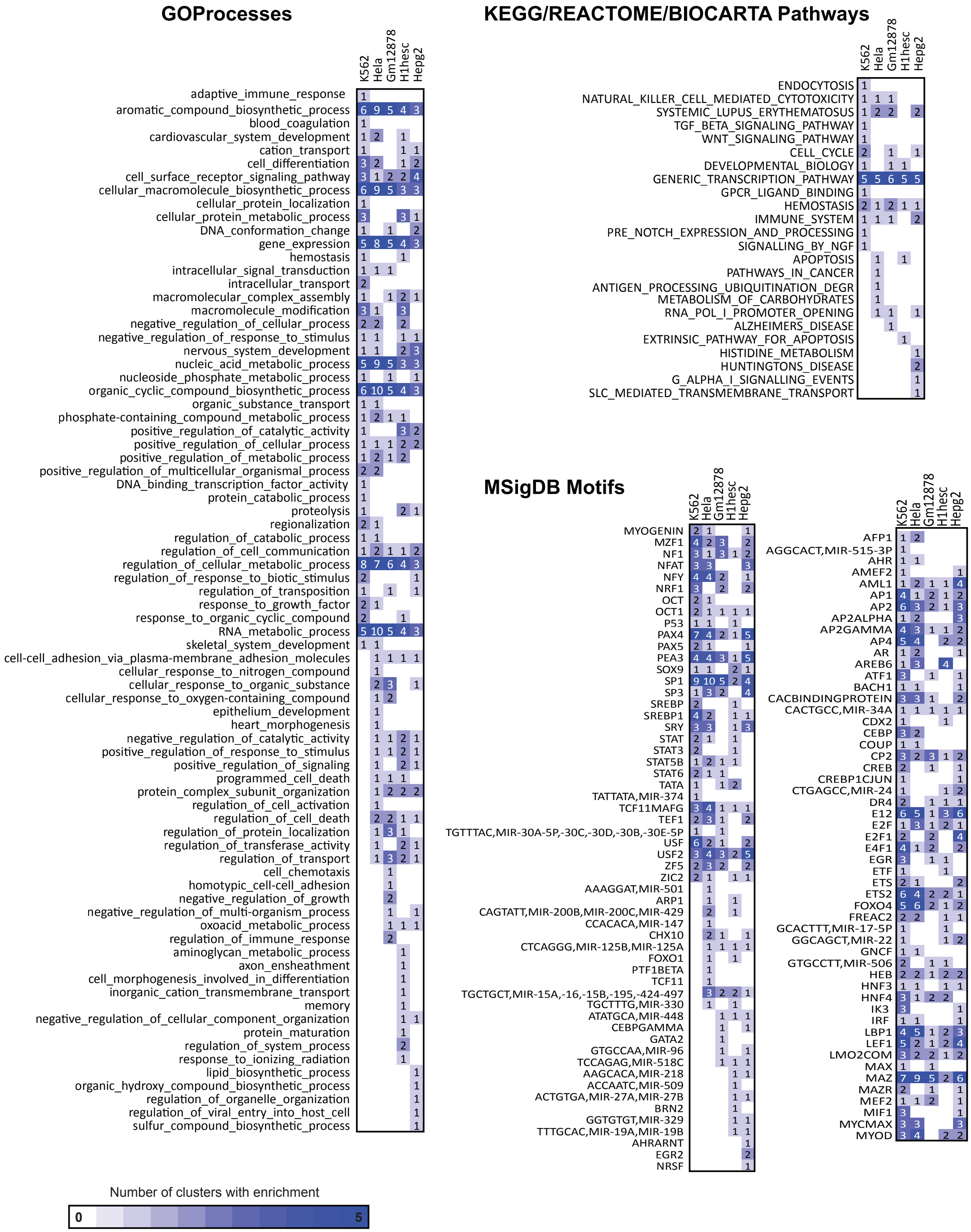

Supplementary Figure 8. Shown are the GO, curated pathways from MSigDB, and transcription factor and microRNA motifs from MSigDB enriched in Multi-output and Multi-input Multi-output enhancer-promoter subnetworks with at least 5 genes. The blue intensity corresponds to the number of clusters enriched for the term. Terms were selected such that they were the most significant term (FDR corrected Hypergeometric P-value <0.05) in at least one subnetwork in one of the cell lines.

Supplementary Figure 9

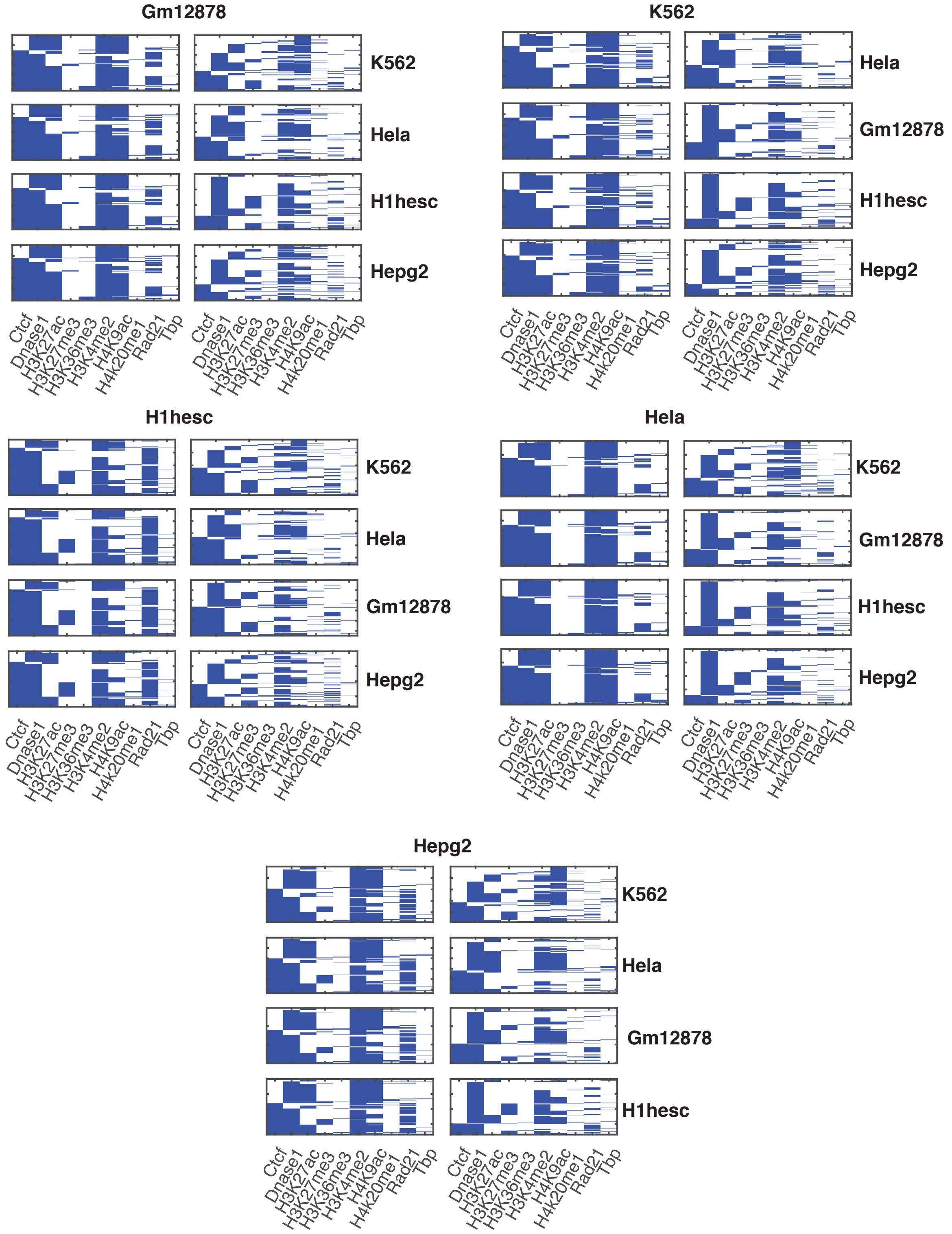

Supplementary Figure 9. Shown are the different regulatory signals (blue: present, white: absent) for enhancers contributing to cell-line specific interactions when comparing pairs of cell lines. The rows in each heat map are ordered in the same way as **Fig 6F**. The cell line specified on top of each set of eight heat maps is the cell line in which enhancers interact. The left set of heatmaps (all identical for a cell line) are associated with the feature presence absence in the cell line listed on top. The right set of heat maps are the features for enhancers in cell lines listed on the side with each heat map.

Supplementary Method for Feature refinement

Although both Group Lasso and Random Forests gave us feature rankings, they by themselves did not solve our feature selection problem because the features selected by Group Lasso had a significantly lower performance, while those by Random Forests were too many. Furthermore, the features tended to be correlated (**Supplementary Fig 3**), and are also known to function together, but none of these feature selection approaches exploited the correlation among features. To refine features we started with the intersection the Group Lasso and Random Forests feature ranking-based important features. and used the correlation among features to inform our feature refinement. For example, CTCF, SMC3, and RAD21 were highly correlated in all four cell lines. Similarly, H3K4me3 and H3K4me2 were highly correlated with each other and with H3K9ac and H3K27ac. H3K36me3, H3K79me2 were correlated in K562, Hela, and moderately in Gm12878. We first eliminated one feature at a time, and found that removal of some features did not result in significant loss in performance, but did so when removed in combination with other features. Based on this H3K4me3 was removed. Between H3K36me3 and H3K79me2, while H3K79me2 could be removed H3K36me3 and H3K79me2 removal together resulted in significant loss in performance. Between SMC3, RAD21 and CTCF, we found individual datasets did not change performance, but removal of RAD21 and CTCF together resulted in significant loss in performance. Finally, we considered triplet feature removals where we excluded H3K4me3, SMC3, H3K9ac, and H3K4me3, SMC3, H3K27ac, and H3K4me3, SMC3, H3K79me2. Removals including H3K9ac resulted in significant loss in performance, and therefore we continued with H3K4me3, SMC3 and either keeping H3K79me2 or H3K9ac. Elimination of features resulted in two feature sets that we wished to expand by adding additional features identified from Random Forests. These included the Group Lasso intersection set that excluded H3K4me3, SMC3 and either, H3K79me2 or H3K27ac, referred here as noK27ac_K4me3_Smc3 and noK79me2_K4me3_Smc3 respectively. The RF features we considered were H4K20me1, CMYC, JUND, USF2 and TBP. We hypothesized that the inability of a classifier to predict interactions is due to the lack of DNA binding factors. We considered individual feature additions as well pairs of feature additions that combined H4K20me1 with one of the transcription factors.

We found that adding H4K20me1 with TBP or JUND while excluding H3K79me2, H3K4me3 and SMC3

(noK79me2_K4me3_Smc3 feature set, **Supplementary Fig 4**) gave the best performance and was close to using the Random Forests based feature set. We also noticed that addition of TBP or JUND alone was not sufficient to improve performance suggesting that H4K20me1 was a required feature in the dataset. Finally, we asked whether SMC3 could replace the general transcription factors by adding it back with H4K20me1 to noK27ac_K4me3_SMC3 feature set with H4K20me1 and to the noK79me2_K4me3_Smc3 set but this was not able to outperform the noK79me2_K4me3_Smc3 with TBP or JUND. We selected H3K27ac over H3K79me2 because H3K27ac is know be associated with enhancer regions and H3K79me2 is correlated with H4K20me1 and H3K36me3.
